# Efficacy and safety of acupuncture-related therapies in improving insulin resistance, reproductive endocrine outcomes, and ovarian morphology in polycystic ovary syndrome: a systematic review and network meta-analysis

**DOI:** 10.3389/fendo.2026.1748814

**Published:** 2026-02-27

**Authors:** Zhenping Du, Mamuke· Yerebake, Anqi Shi, Shan Zhao, Shutong Li, Yu Wan, Jun Wang, Chaoqun Yan

**Affiliations:** 1 Department of Acupuncture, Dongzhimen Hospital, Beijing University of Chinese Medicine, Beijing, China; 2 Department of Gynaecology, Dongzhimen Hospital, Beijing University of Chinese Medicine, Beijing, China

**Keywords:** acupuncture-related therapies, network meta-analysis, NMA, polycystic ovary syndrome, PCOS, systematic review

## Abstract

**Objective:**

This network meta-analysis aimed to compare and rank the efficacy and safety of acupuncture-related therapies (ARTs) for polycystic ovary syndrome (PCOS) in improving insulin resistance (IR), reproductive endocrine outcomes, and ovarian morphology.

**Methods:**

Randomized controlled trials (RCTs) in Chinese and English were retrieved up to September 2025 from eight databases (the Cochrane Library, Web of Science, PubMed, Embase, VIP, CNKI, Wanfang, and CBM). Eligible participants were women with PCOS diagnosed using established international or Chinese criteria. Interventions compared ARTs (e.g., acupuncture, moxibustion, electroacupuncture) versus conventional medication and/or placebo. The primary outcome was homeostatic model assessment of IR (HOMA-IR). Secondary outcomes included fasting insulin (FINS), fasting blood glucose (FBG), body mass index (BMI), waist-to-hip ratio (WHR), testosterone (T), luteinizing hormone (LH), follicle-stimulating hormone (FSH), LH/FSH, antral follicle count (AFC), and ovarian volume (OV). Risk of bias was assessed using Review Manager 5.3, and network meta-analysis with surface under the cumulative ranking curve (SUCRA) rankings was conducted in Stata 17.0. All outcomes were summarized as mean differences (MDs) with 95% confidence intervals (CIs).

**Results:**

53 RCTs involving 4,406 participants and 12 ART regimens (including two combined regimens) were included. Acupoint injection therapy (AIT) and acupuncture plus moxibustion (Acu + Moxi) significantly reduced HOMA-IR (MD = 2.20, 95% CI 0.44-3.96; MD = 1.06, 95% CI 0.28-1.84). AIT, catgut implantation at acupoint (CIAA), and Acu reduced FINS (MD = 7.30, 95% CI 0.83-13.77; MD = 3.11, 95% CI 1.97-4.25; MD = 2.97, 95% CI 1.87-4.06). Acu + Moxi reduced BMI (MD = 5.80, 95% CI 3.38-8.22), and electroacupuncture (EA) reduced WHR (MD = 0.06, 95% CI 0.02-0.09). Laser acupuncture (LA) reduced T and LH (MD = 0.59, 95% CI 0.33-0.85; MD = 3.00, 95% CI 0.47-5.53). For ovarian morphology, warm needle therapy (WNT) and Acu reduced AFC (MD = 4.08, 95% CI 0.63-7.53; MD = 3.06, 95% CI 1.07-5.05), and Acu reduced ovarian volume (OV) (MD = 2.38, 95% CI 0.67-4.08). Overall, Acu ranked among the top interventions across multiple outcomes. Most reported adverse events were non-serious and transient. Adverse-event reporting was limited across trials.

**Conclusion:**

ARTs may be safe and effective complementary therapies for improving IR, reproductive endocrine outcomes, and ovarian morphology in women with PCOS.

**Systematic Review Registration:**

https://www.crd.york.ac.uk/PROSPERO/view/CRD420251151249, identifier CRD420251151249.

## 1 Introduction

Polycystic Ovary Syndrome (PCOS) is a common endocrine metabolic disorder among women of reproductive age. Its global prevalence is approximately 8% to 15% (1, 2). First described by Stein and Leventhal in 1935 (3), it is also known as Stein-Leventhal syndrome. PCOS is associated with a broad spectrum of adverse health consequences. These include metabolic abnormalities such as obesity and insulin resistance (IR), increased cardiometabolic risk, and psychological concerns such as mood disturbances and sleep problems. Reproductive manifestations are also common, including ovulatory dysfunction, infertility, and hyperandrogenism (4, 5). In addition, PCOS may have implications for offspring health, and emerging evidence suggests potential associations with neurodevelopmental, metabolic, and reproductive outcomes in the next generation (6).

The pathophysiology of PCOS is complex and multifactorial. IR is a key feature of PCOS and is closely linked to both metabolic disturbances and reproductive dysfunction (7, 8). Metabolic dysfunction characterized by IR and compensatory hyperinsulinemia is common in affected individuals (4). Notably, IR has been reported in 35%-80% of women with PCOS, regardless of obesity status; this wide range likely reflects differences in diagnostic criteria, populations, and IR assessment methods, yet IR severity is consistently higher than in non-PCOS controls (9, 10). Moreover, prospective evidence supports an association between PCOS and type 2 diabetes mellitus risk (11), underscoring the clinical importance of improving IR-related outcomes in PCOS management.

Clinical management of PCOS typically begins with lifestyle interventions, including weight management, physical activity, and dietary modification. Pharmacological options are selected according to the patient’s primary concerns, such as reproductive dysfunction, hyperandrogenism, or metabolic risk, and may include oral contraceptives or insulin-sensitizing agents. However, symptom control may be incomplete, and long-term adherence can be limited by adverse effects or patient preferences (12–15). Therefore, safe and effective adjunctive therapies remain of clinical interest, particularly for metabolic risk reduction and reproductive endocrine regulation.

Acupuncture-related therapies (ARTs) are widely used complementary approaches and have been applied in reproductive endocrinology and infertility, including PCOS (16). Experimental studies suggest that acupuncture may modulate sex hormone profiles and improve ovarian morphology in PCOS models, with potential downstream effects on reproductive and metabolic endocrine function (17, 18). Meanwhile, randomized controlled trials (RCTs) of ARTs for PCOS have increased over recent years, and several systematic reviews have suggested potential benefits and acceptable safety profiles (19, 20). However, existing evidence is heterogeneous in intervention modalities, treatment parameters, and outcome reporting, and most prior syntheses are limited to pairwise comparisons or focus on selected reproductive outcomes.

Although ARTs may be promising for PCOS, there remains no clear comparative hierarchy across different ART modalities regarding improvements in IR-related indicators and reproductive endocrine outcomes, nor is the evidence for ovarian morphology outcomes synthesized in a way that supports comparative decision-making. Currently published systematic reviews commonly compare a single ART modality against one control, or emphasize sex hormones and clinical symptoms, leaving uncertainty about the relative effectiveness of different ARTs on key metabolic and reproductive endpoints.

Network meta-analysis (NMA) provides a methodological framework for the comparative evaluation of multiple interventions (21). In this study, we conducted a NMA to compare the efficacy and safety of ARTs for PCOS, focusing on IR and related metabolic outcomes, reproductive endocrine outcomes, and ovarian morphology indicators. The results aim to inform clinical decision-making regarding the comparative effects of different ART modalities.

## 2 Materials and methods

This NMA was conducted following the Preferred Reporting Items for Systematic Reviews and Meta-Analyses (PRISMA) guidelines (**Supplementary Table S1**) and has been registered on the International Prospective Register of Systematic Reviews (PROSPERO) (registration number: CRD420251151249).

### 2.1 Search strategy

To comprehensively collect relevant studies, systematic searches were conducted across the following databases: Cochrane Library, Web of Science, PubMed, Embase, China Science and Technology Journal Database (VIP), China National Knowledge Infrastructure (CNKI), Wanfang Database (WF), and China Biomedical Literature Database (CBM). The search period spanned from the inception of each database up to 6 September 2025. The search strategy combined Medical Subject Headings (MeSH) and free-text terms, restricted to publicly published studies. Specific search terms are presented in **Table 1** (using PubMed as an example), while **Supplementary Table S2** provides the complete search strategies for each database.

**Table 1:** Search strategy (through PubMed).

ID	Search terms	Results
#1	Polycystic Ovary Syndrome [MeSH Terms]	20358
#2	(((((((((((((Ovary Syndrome, Polycystic[Title/Abstract])) OR (Syndrome, Polycystic Ovary[Title/Abstract])) OR (Polycystic Ovarian Syndrome[Title/Abstract])) OR (Ovarian Syndrome, Polycystic[Title/Abstract])) OR (Sclerocystic Ovarian Degeneration[Title/Abstract])) OR (Ovarian Degeneration, Sclerocystic[Title/Abstract])) OR (Sclerocystic Ovary Syndrome[Title/Abstract])) OR (Stein-Leventhal Syndrome[Title/Abstract])) OR (Stein Leventhal Syndrome[Title/Abstract])) OR (Syndrome, Stein-Leventhal[Title/Abstract])) OR (Sclerocystic Ovaries[Title/Abstract])) OR (Ovary, Sclerocystic[Title/Abstract])) OR (Sclerocystic Ovary[Title/Abstract])	5553
#3	#1 OR #2	22329
#4	(((((Acupuncture [MeSH Terms]) OR (Acupuncture Therapy [MeSH Terms])) OR (Acupuncture, Ear [MeSH Terms])) OR (Acupuncture Points [MeSH Terms])) OR (Electroacupuncture [MeSH Terms])) OR (Moxibustion [MeSH Terms])	33226
#5	((((((((((((((((((Pharmacopuncture[Title/Abstract]) OR (Acupuncture Treatment*[Title/Abstract])) OR (Treatment, Acupuncture[Title/Abstract])) OR (Therapy, Acupuncture[Title/Abstract])) OR (Pharmacoacupuncture Treatment[Title/Abstract])) OR (Treatment, Pharmacoacupuncture[Title/Abstract])) OR (Pharmacoacupuncture Therapy[Title/Abstract])) OR (Therapy, Pharmacoacupuncture[Title/Abstract])) OR (Acupotomy[Title/Abstract])) OR (Acupotomies[Title/Abstract])) OR (Acupunctures, Ear[Title/Abstract])) OR (Ear Acupunctures[Title/Abstract])) OR (Acupuncture*, Auricular[Title/Abstract])) OR (Auricular Acupuncture*[Title/Abstract])) OR (Ear Acupuncture[Title/Abstract])) OR (Acupuncture Point[Title/Abstract])) OR (Point*, Acupuncture[Title/Abstract])) OR (Acupoint*[Title/Abstract])) OR (Electroacupuncture[Title/Abstract])	19455
#6	#4 OR #5	37823
#7	#3 AND #6	225

### 2.2 Inclusion and exclusion criteria

#### 2.2.1 Type of studies

Published RCTs on ARTs for the treatment of PCOS, limited to Chinese or English language publications.

#### 2.2.2 Type of participants

Patients included must have a diagnosis of PCOS based on one of the following (1): Rotterdam Criteria from the European Society of Human Reproduction and Embryology and the American Society for Reproductive Medicine (January 2003) (7); (2) Chinese Diagnostic and Treatment Guidelines for PCOS (22); or (3) PCOS-related criteria in Obstetrics and Gynaecology (23).

#### 2.2.3 Type of interventions

The observation group utilized at least one ART or a combination of two or more ARTs regimen. The control group received conventional medication (oral herbal or Western pharmaceuticals) and/or placebo. Both groups faced no restrictions regarding treatment sites, duration, needles, materials, drug formulations, or dosages.

#### 2.2.4 Type of outcome indicator

Eligible trials were required to report at least one IR-related outcome. Primary outcome measure: homeostatic model assessment of insulin resistance (HOMA-IR).Secondary outcome measures include: (a) fasting insulin (FINS); (b) fasting blood glucose (FBG); (c) body mass index (BMI); (d) waist-to-hip ratio (WHR); (e) testosterone (T); (f) luteinizing hormone (LH); (g) follicle-stimulating hormone (FSH); (h) LH/FSH ratio; (i) ovarian volume (OV); and (j) antral follicle count (AFC).

Additional outcomes of interest were extracted when reported, including Estradiol (E_2_) and oral glucose tolerance test (OGTT) indices. Due to sparse data and substantial heterogeneity in measurement timing and units, OGTT and E_2_ were summarized descriptively rather than synthesized in the network meta-analysis (see **Supplementary Tables S6.12**, **S6.13**).

#### 2.2.5 Exclusion criteria

The exclusion criteria for studies are as follows: (a) duplicate publications; (b) abstracts or conference proceedings; (c) non-peer-reviewed literature; (d) non-randomized controlled studies; and (e) articles where full text or data were unavailable.

### 2.3 Data extraction and quality evaluation

Two researchers (Du and Mamuke) independently extracted data. They then cross-checked and validated the results. Discrepancies were resolved through discussion or by consulting an independent third party (Wang) to reach a consensus. Extracted data included: (a) basic information; (b) participant baseline characteristics and treatment methods, including electroacupuncture parameters when applicable (frequency/waveform/intensity when reported, session duration, and total course; see **Supplementary Table S4.1**); (c) key elements for risk of bias assessment; and (d) outcome measures and corresponding data.

### 2.4 Risk of bias of the included studies

Du and Mamuke independently assessed the risk of bias in the included RCTs and cross-checked their assessments for consistency. When they disagreed, Wang re-evaluated the studies. They used the tools recommended in the Cochrane Handbook for Systematic Reviews (Version 5.1.0).

### 2.5 Statistical analysis

This study employed Stata 17.0 software and its network package to conduct NMA. All continuous outcomes were summarized as mean differences (MDs) with 95% confidence intervals (CIs). Since lower values indicate improvement for all outcomes in this review, effect directions were aligned so that positive MDs consistently favored ARTs. The overall consistency of the evidence network was verified using a global inconsistency model (When P > 0.05, it indicates that global inconsistency is not significant; the consistency model was ultimately adopted throughout this study.) Local inconsistency was evaluated using node splitting (P < 0.05 indicating local inconsistency). Loop inconsistency was analyzed by calculating the 95% CI for the inconsistency factors (IF) using the ifplot command (a zero inclusion indicates no significant loop inconsistency). Simultaneously, the surface under the cumulative ranking curve (SUCRA) was calculated for each intervention, with pairwise comparisons presented in a league table to rank the probability of intervention effectiveness. SUCRA values (0%–100%) probabilistically rank intervention effectiveness, with higher values indicating a greater likelihood of being the optimal treatment. The methodological quality and risk of bias of included studies were assessed using Rev Man 5.3 software, as recommended by the Cochrane Collaboration.

## 3 Results

### 3.1 Included articles

**Figure 1:**
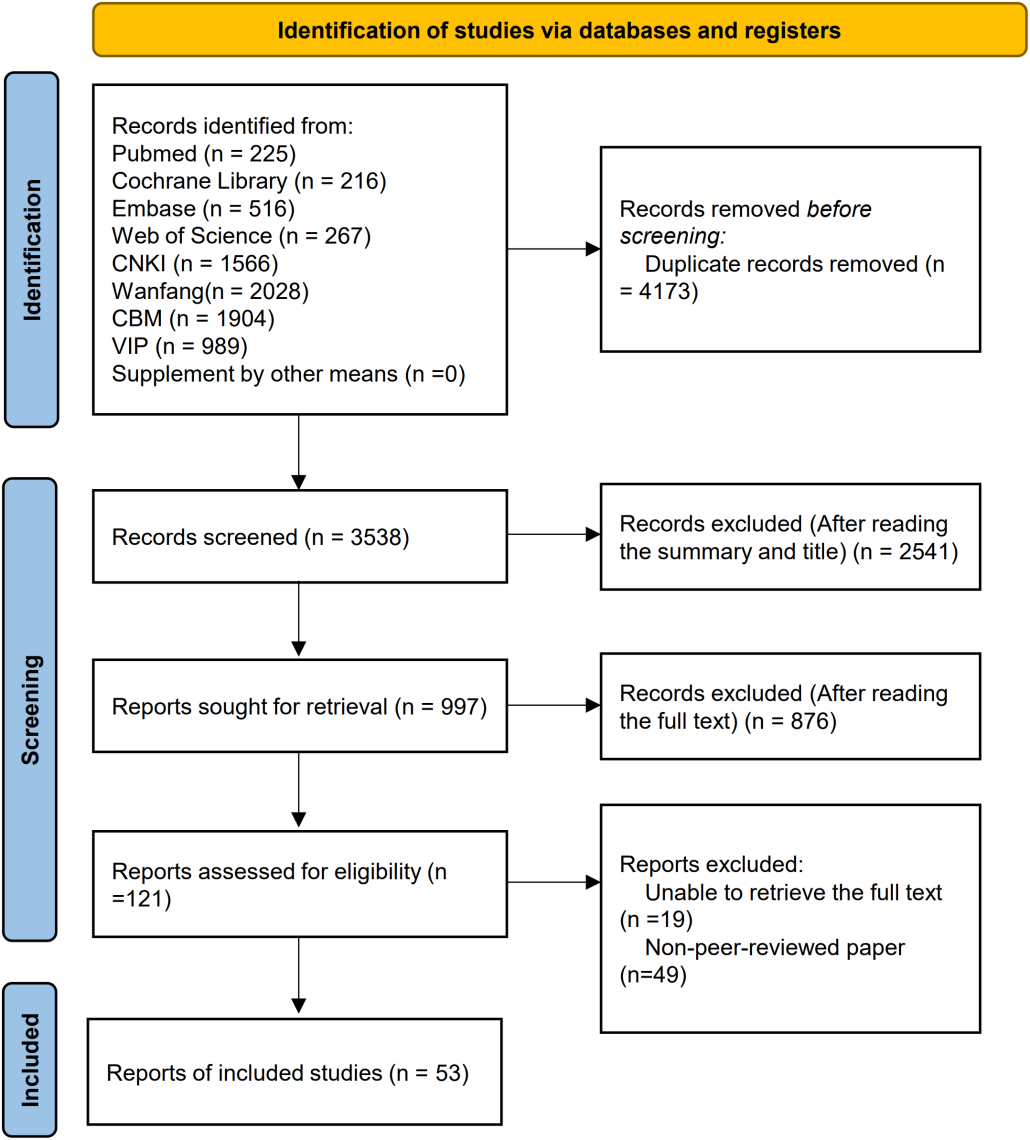
Literature screening process and results.

The initial search across multiple databases yielded 7,711 relevant articles, including those from the Cochrane Library (n = 216), Web of Science (n = 267), PubMed (n = 225), Embase (n = 516), VIP (n = 989), CNKI (n = 1,566), WF (n = 2,028), and CBM (n = 1,904). Following multiple rounds of rigorous screening, 53 studies (24–76) were ultimately included in this NMA. These studies were published between 2007 and 2025. A detailed literature screening flowchart and results are presented in **Figure 1**.

### 3.2 Study characteristics

**Figure 2:**
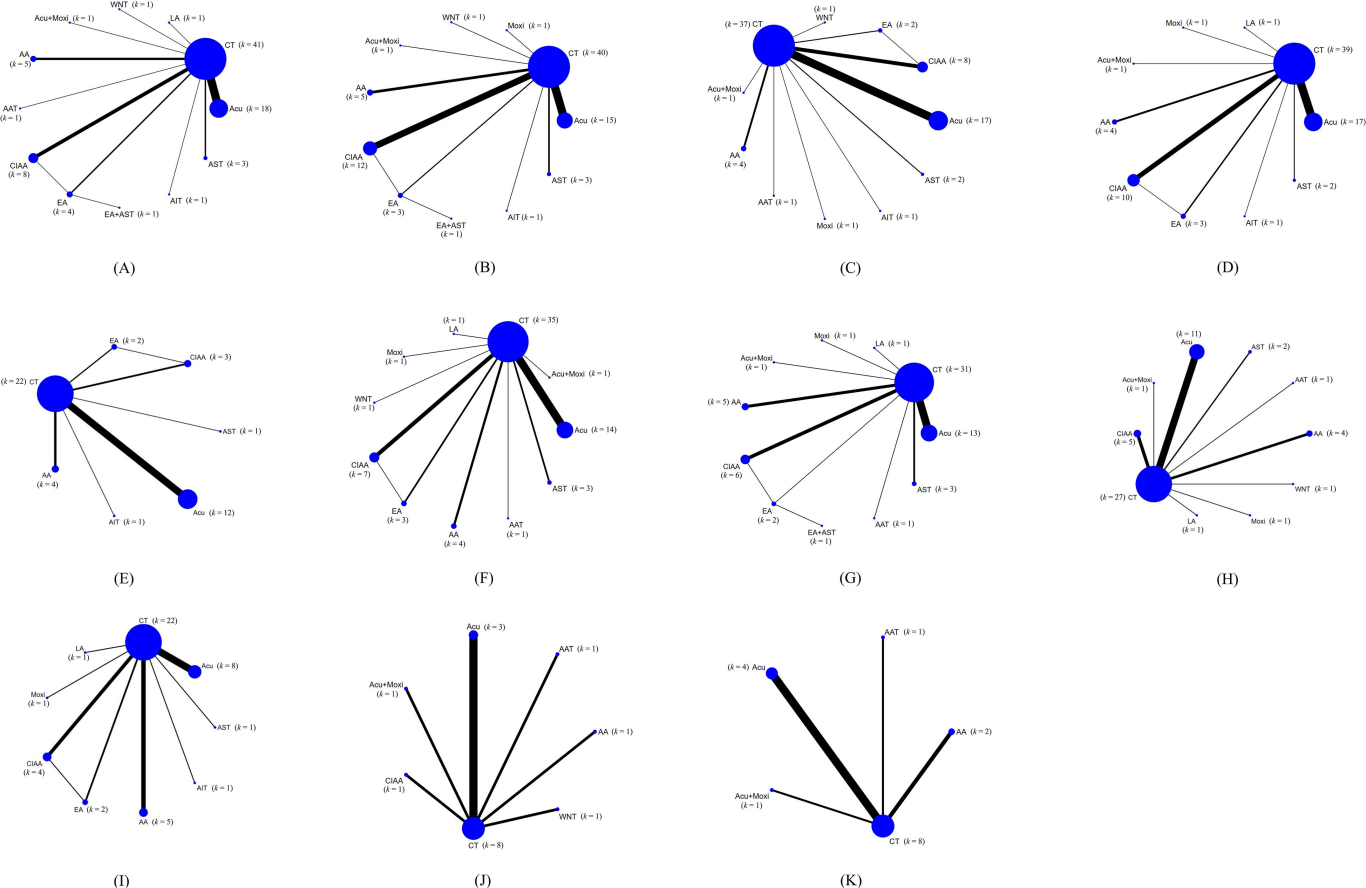
Evidence network graphs of all studies on different outcome measures. **(A)** homeostatic model assessment of insulin resistance (HOMA-IR); **(B)** fasting insulin (FINS); **(C)** fasting blood glucose (FBG); **(D)** body mass index (BMI); **(E)** waist-to-hip ratio (WHR); **(F)** testosterone **(T)**; **(G)** LH (luteinizing hormone); **(H)** FSH (follicle-stimulating hormone); **(I)** LH/FSH; **(J)** antral follicle count (AFC); **(K)** ovarian volume (OV). Line thickness indicates study count per direct comparison; node size shows the number of studies contributing to each intervention (k). Lines represent direct head-to-head comparisons; line crossings are only a visual overlap from the plotting layout and do not represent any special relationship. Node labels indicate (k) CT, conventional treatment; Acu, acupuncture; LA, laser acupuncture; AA, abdominal acupuncture; EA, electroacupuncture; CIAA, catgut implantation at acupoint; WNT, warm needle acupuncture; AST, auricular seed therapy; AAT, acupoint application therapy; Moxi, moxibustion; AIT, acupoint injection therapy.

This NMA included 53 studies involving 4,406 patients, all of which were RCTs (24–76). The studies encompassed 11 distinct treatment modalities: conventional treatment (CT), acupuncture (Acu), laser acupuncture (LA), abdominal acupuncture (AA), electroacupuncture (EA), catgut implantation at acupoint (CIAA), warm needle acupuncture (WNT), auricular seed therapy (AST), acupoint application therapy (AAT), moxibustion (Moxi), acupoint injection therapy (AIT). In addition to single-modality ARTs, some trials evaluated combined ART regimens (e.g., Acu + Moxi and EA + AST), which were treated as separate nodes in the network when applicable. **Supplementary Table S3** details the abbreviations and definitions for the different therapies. **Supplementary Table S4** provides further details on the included RCTs. A comprehensive network diagram illustrating all comparisons of outcomes is presented in **Figure 2**.

### 3.3 Literature quality evaluation

**Figure 3:**
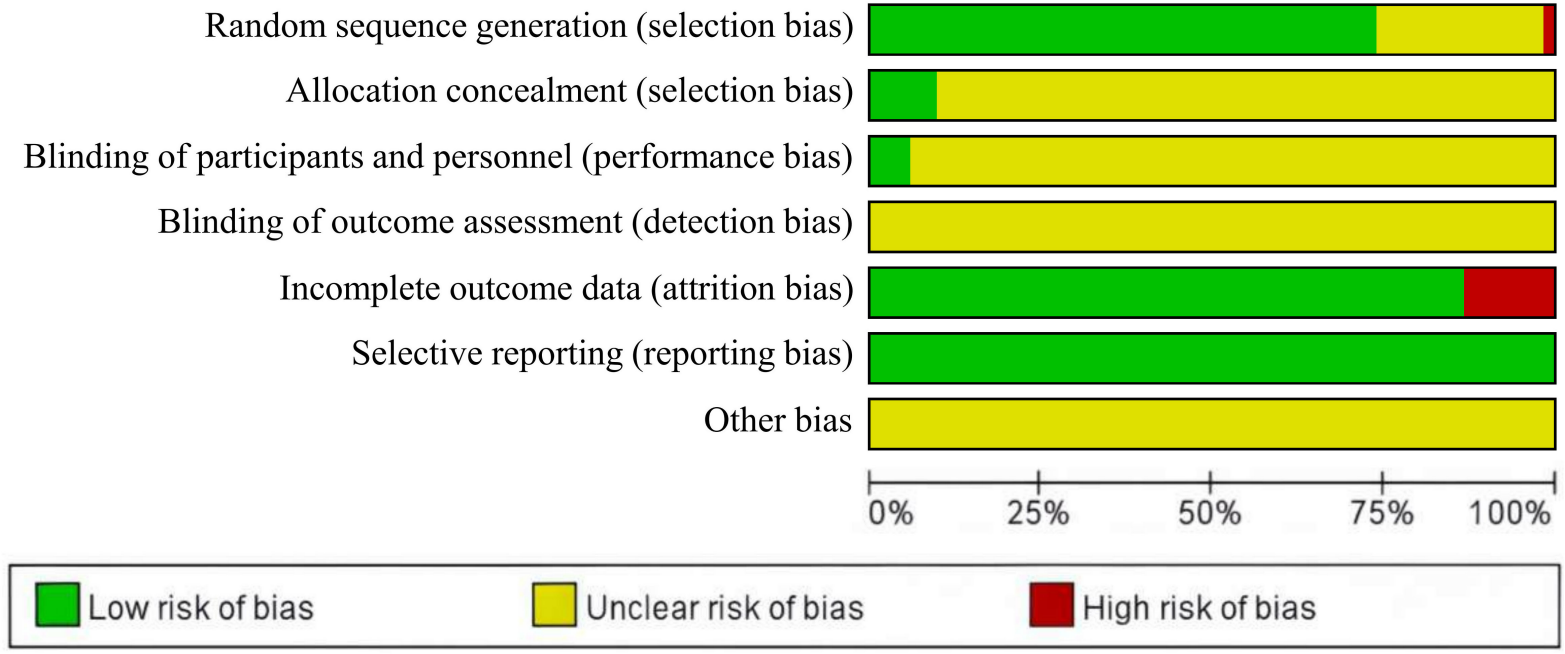
Risk of bias assessment.

This study included 53 RCTs and conducted a comprehensive quality assessment. Regarding methods for random sequence generation, one study (31) allocated patients according to the order of diagnosis after admission, potentially introducing selection bias, and was therefore judged to be at high risk. Fourteen studies (33, 36, 38, 43, 44, 48–50, 58, 61, 65, 67, 74, 75) mentioned randomization but failed to describe the randomization method, and were therefore categorized as having an unclear risk. Thirty-eight studies (24–30, 32, 34, 35, 37, 39–42, 45–47, 51–57, 59, 60, 62–64, 66, 68–73, 76) were considered low risk as they employed random number tables or computer-based random sequence generators for subject allocation. Regarding allocation concealment, 5 studies (24–28) were low risk, whilst all remaining studies (29–76) failed to clearly describe their allocation concealment methods, resulting in an unclear risk rating. Only three studies (25, 27, 28) implemented blinding for both subjects and investigators and were assessed as low risk, whilst the remaining studies (24, 26, 29–76) made no mention of blinding and were rated as having unclear risk of bias. Regarding data integrity, seven studies (32, 34, 49, 51, 53, 66, 76) were deemed high risk for failing to explicitly analyze and report reasons for and data from patients who withdrew. All remaining studies (24–31, 33, 35–48, 50, 52, 54–65, 67–75) retained complete datasets. The presence of selective reporting bias was determined by verifying whether these methods were consistent with the reported outcomes. All studies (24–76) fully reported their findings and were thus considered low risk in this regard. All studies (24–76) were of unclear risk for other potential sources of bias and were therefore classified as unclear risk. **Figure 3** displays the results of the bias risk assessment conducted using Rev Man 5.3. Specific details of the evaluation are provided in **Supplementary Table S5**.

### 3.4 Consistency test

The results of global inconsistency tests for all study outcome measures showed P > 0.05, indicating no significant inconsistency across the overall research. The inconsistency test results are presented in detail at the bottom of **Supplementary Figures S1**-**S11**. Local inconsistency tests employing node splitting yielded P > 0.05 across all analyses, indicating no significant local inconsistency. Detailed results are presented in **Supplementary Table S7**. Analysis of loop consistency revealed that the 95% confidence intervals for all loop IF encompassing closed-loop studies contained zero, suggesting non-significant loop inconsistency. Detailed results are presented in **Supplementary Table S8**.

### 3.5 Outcome indicators

**Figure 4:**
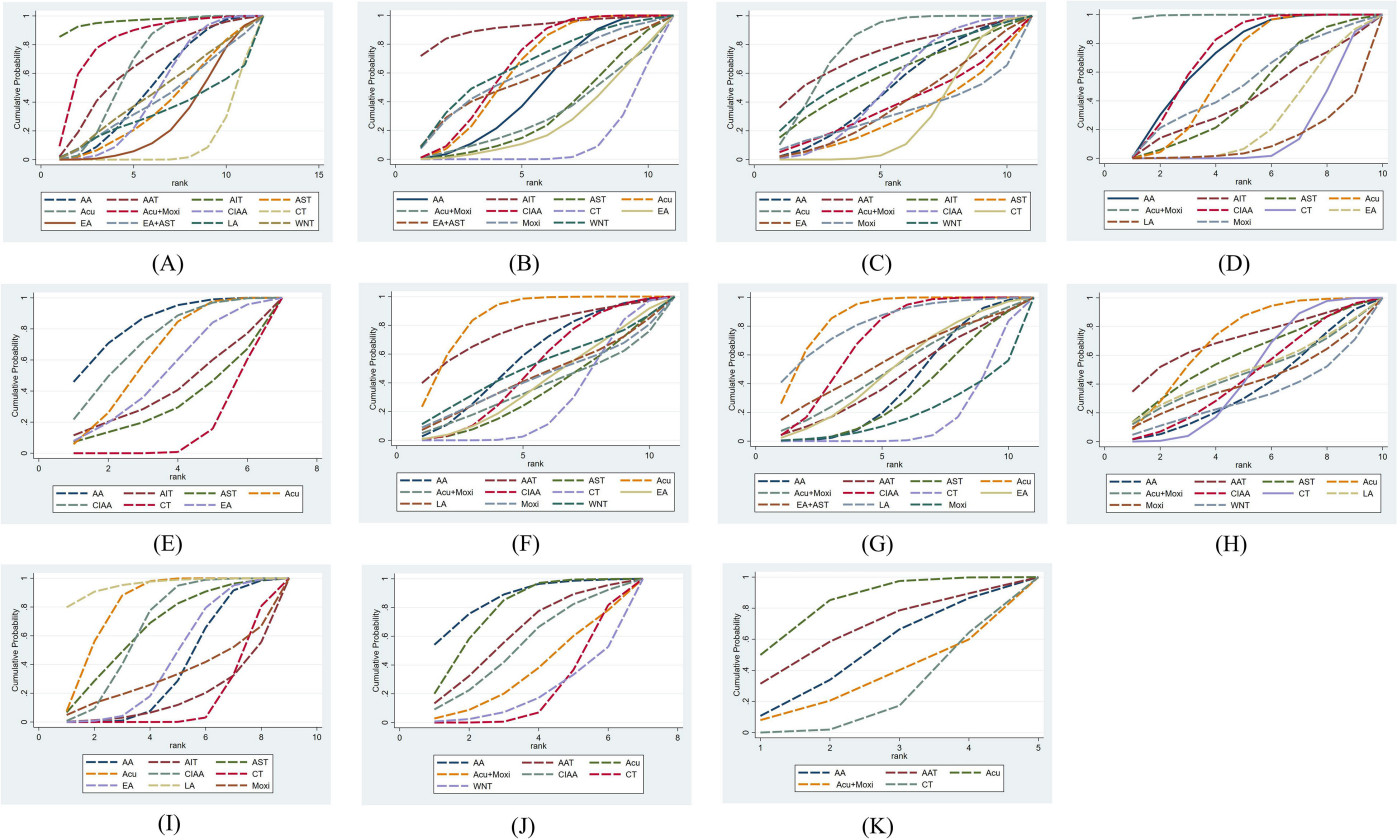
SUCRA rankings of all studies on different outcome measures. **(A)** homeostatic model assessment of insulin resistance (HOMA-IR); **(B)** fasting insulin (FINS); **(C)** fasting blood glucose (FBG); **(D)** body mass index (BMI); **(E)** waist-to-hip ratio (WHR); **(F)** testosterone **(T)**; **(G)** LH (luteinizing hormone); **(H)** FSH (follicle-stimulating hormone); **(I)** LH/FSH; **(J)** antral follicle count (AFC); **(K)** ovarian volume (OV). CT, conventional treatment; Acu, acupuncture; LA, laser acupuncture; AA, abdominal acupuncture; EA, electroacupuncture; CIAA, catgut implantation at acupoint; WNT, warm needle acupuncture; AST, auricular seed therapy; AAT, acupoint application therapy; Moxi, moxibustion; AIT, acupoint injection therapy.

Effect estimates and SUCRA rankings for all outcomes are presented in **Table 2** and **Figure 4**. Detailed pairwise comparisons (league tables) are provided in **Supplementary Tables S6.1**-**S6.11**.

**Table 2:** The SUCRA values of each treatment modality.

Treatment	CT	Acu	LA	AA	EA	CIAA	WNT	EA+AST	AAT	Acu+Moxi	AIT	AST	Moxi
HOMA-IR	9.6	64.7	33.3	50.2	28.2	46.2	45.7	40.6	64.9	82.2	96.2	38	
Rank	12	4	10	5	11	6	7	8	3	2	1	9	
FINS	10.8	62.8		49.5	25.2	65.7	65.2	56.4		30.7	91.5	31.7	60.4
Rank	11	4		7	10	2	3	6		9	1	8	5
FBG	28.4	79.6		49.4	38.9	52.1	64.6		73.9	40.4	58.7	30	34
Rank	11	1		6	8	5	3		2	7	4	10	9
BMI	16.7	61.8	11.5	71.5	26.4	73.2				99.6	41.8	45.2	52.4
Rank	9	4	10	3	8	2				1	7	6	5
WHR	12.6	61.6		83	50.8	71.3					40.1	30.6	
Rank	7	3		1	4	2					5	6	
T	28.3	85.7	45	58.8	40.6	49.7	51.1		76.1	36.2		35	43.5
Rank	11	1	6	3	8	5	4		2	9		10	7
LH	15	86.9	82.3	40	50.6	71.1		57.2	44.2	50.4		33.3	19.1
Rank	11	1	2	8	5	3		4	7	6		9	10
FSH	46.1	71.5	49.1	37.1		45.1	31.1		72.1	46.6		60.9	40.4
Rank	6	2	4	9		7	10		1	5		3	8
LH/FSH	14.7	81.1	94.9	36.7	43.9	65.3					16.2	65.1	32.2
Rank	9	2	1	6	5	3					8	4	7
AFC	20.9	76.1		85.3		52.7	18.8		60.9	35.2			
Rank	6	2		1		4	7		3	5			
OV	20.5	82.7		50.2					64.3	32.4			
Rank	5	1		3					2	4			

CT, conventional treatment; Acu: acupuncture; LA, laser acupuncture; AA, abdominal acupuncture; EA, electroacupuncture; CIAA, catgut implantation at acupoint; WNT, warm needle acupuncture; AST, auricular seed therapy; AAT, acupoint application therapy; Moxi, moxibustion; AIT, acupoint injection therapy.

#### 3.5.1 HOMA-IR

42 studies (n=3,570) contributed to the network (24–26, 28–32, 34–40, 42, 45–47, 49–53, 55–57, 59, 60, 62–64, 66, 67, 69, 70, 72–76). Compared with conventional treatment, AIT (MD = 2.20, 95% CI 0.44-3.96) and Acu + Moxi (MD = 1.06, 95% CI 0.28-1.84) showed statistically significant reductions in HOMA-IR. In the ranking analysis, AIT had the highest probability of being the most effective intervention (SUCRA 96.2%), followed by Acu + Moxi (82.2%), while AAT and Acu formed a second tier (64.9% and 64.7%). Detailed pairwise estimates are provided in **Supplementary Table S6.1**.

#### 3.5.2 FINS

41 studies (n=3,213) contributed to the network (24, 27, 29–39, 41, 44–52, 54–59, 61–67, 69–73). Compared with conventional treatment, AIT (MD = 7.30, 95% CI 0.83-13.77), CIAA (MD = 3.11, 95% CI 1.97-4.25), and Acu (MD = 2.97, 95% CI 1.87-4.06) showed statistically significant reductions in FINS. In the ranking analysis, AIT had the highest probability of being the most effective intervention (SUCRA 91.5%), followed by CIAA (65.7%) and WNT (65.2%), while Acu ranked next (62.6%). Detailed pairwise estimates are provided in **Supplementary Table S6.2**.

#### 3.5.3 FBG

37 studies (n=2,896) contributed to the network (24, 27, 29–38, 40, 41, 44–52, 54–56, 59, 62, 64–70, 72, 74). Compared with conventional treatment, Acu (MD = 0.56, 95% CI 0.31-0.81) showed a statistically significant reduction in FBG. In the ranking analysis, Acu had the highest probability of being the most effective intervention (SUCRA 79.6%), followed by AAT (73.9%) and WNT (64.6%), while AIT ranked next (58.7%). Detailed pairwise estimates are provided in **Supplementary Table S6.3**.

#### 3.5.4 BMI

39 studies (n=2,985) contributed to the network (25–27, 29, 31–34, 36–38, 42–51, 53–56, 58, 60, 61, 63–65, 67–70, 72–75). Compared with conventional treatment, Acu+Moxi (MD = 5.80, 95% CI 3.38-8.22), Acu (MD = 2.29, 95% CI 1.57-3.01), and EA (MD = 2.28, 95% CI 1.00-3.55) showed statistically significant reductions in BMI. In the ranking analysis, Acu + Moxi had the highest probability of being the most effective intervention (SUCRA 99.6%), followed by CIAA (73.2%) and AA (71.5%), while Acu ranked next (61.8%). Detailed pairwise estimates are provided in **Supplementary Table S6.4**.

#### 3.5.5 WHR

22 studies (n=2,639) contributed to the network (26, 29, 31, 32, 34, 37, 38, 42–44, 47, 53, 55, 56, 60, 63, 65, 68, 69, 72, 74, 75). Compared with conventional treatment, EA (MD = 0.06, 95% CI 0.02-0.09) and Acu (MD = 0.05, 95% CI 0.01-0.08) showed statistically significant reductions in WHR. In the ranking analysis, AA had the highest probability of being the most effective intervention (SUCRA 83.0%), followed by CIAA (71.3%), while Acu ranked next (61.6%). Detailed pairwise estimates are provided in **Supplementary Table S6.5**.

#### 3.5.6 T

35 studies (n=2,898) contributed to the network (25, 29–31, 33, 35, 36, 38, 40, 41, 43–47, 49, 51, 52, 54, 56, 57, 59, 60, 63–67, 69–72, 74–76). Compared with conventional treatment, LA (MD = 0.59, 95% CI 0.33-0.85) showed a statistically significant reduction in T levels. In the ranking analysis, Acu had the highest probability of being the most effective intervention (SUCRA 85.7%), followed by AAT (76.1%), while AA (58.8%) and WNT (51.1%) formed a second tier. Detailed pairwise estimates are provided in **Supplementary Table S6.6**.

#### 3.5.7 LH

32 studies (n=2,639) contributed to the network (24–26, 29, 31, 33, 36, 38–41, 44–47, 49, 51, 54, 56, 57, 59, 63–72, 75). Compared with conventional treatment, LA (MD = 3.00, 95% CI 0.47-5.53), Acu (MD = 2.89, 95% CI 2.12-3.66), and CIAA (MD = 2.18, 95% CI 1.09-3.27) showed statistically significant reductions in LH levels. In the ranking analysis, Acu had the highest probability of being the most effective intervention (SUCRA 86.9%), followed by LA (82.3%), while CIAA (71.1%) and EA+AST (57.2%) formed a second tier. Detailed pairwise estimates are provided in **Supplementary Table S6.7**.

#### 3.5.8 FSH

27 studies (n=2,193) contributed to the network (24–26, 30, 31, 33, 35, 36, 40, 41, 44–47, 49, 51, 54, 57, 59, 65–72). Compared with conventional treatment, reductions in FSH levels were not statistically significant across interventions (all 95% CIs crossed zero). In the ranking analysis, AAT (SUCRA 72.1%) and Acu (71.7%) ranked highest, followed by AST (60.9%); these rankings should be interpreted cautiously given the lack of statistically significant differences. Detailed pairwise estimates are provided in **Supplementary Table S6.8**.

#### 3.5.9 LH/FSH

22 studies (n=1,725) contributed to the network (24–26, 29, 31, 36, 38, 43–47, 52, 54–56, 60, 64, 67, 69, 71, 74). Compared with conventional treatment, LA (MD = 0.70, 95% CI 0.26–1.14) and Acu (MD = 0.43, 95% CI 0.31-0.55) showed statistically significant reductions in the LH/FSH ratio. In the ranking analysis, LA had the highest probability of being the most effective intervention (SUCRA 94.9%), followed by Acu (81.1%), while CIAA (65.3%) and AST (65.1%) formed a second tier. Detailed pairwise estimates are provided in **Supplementary Table S6.9**.

#### 3.5.10 AFC

8 studies (n=851) contributed to the network (30, 35, 40, 44, 49, 57, 64, 71). Compared with conventional treatment, WNT (MD = 4.08, 95% CI 0.63-7.53) and Acu (MD = 3.06, 95% CI 1.07-5.05) showed statistically significant reductions in AFC. In the ranking analysis, AA had the highest probability of being the most effective intervention (SUCRA 85.3%), followed by Acu (76.1%), while AAT (60.9%) and CIAA (52.7%) formed a second tier. Detailed pairwise estimates are provided in **Supplementary Table S6.10**.

#### 3.5.11 OV

8 studies (n=819) contributed to the network (30, 40, 44, 49, 64, 69, 71, 75). Compared with conventional treatment, Acu (MD = 2.38, 95% CI 0.67–4.08) showed a statistically significant reduction in OV. In the ranking analysis, Acu had the highest probability of being the most effective intervention (SUCRA 82.7%), followed by AAT (64.3%), while AA ranked next (50.2%). Detailed pairwise estimates are provided in **Supplementary Table S6.11**.

#### 3.5.12 Exploratory outcomes

E_2_ was reported in 9 trials; however, due to sparse data and substantial heterogeneity in sampling time points and units, together with inconsistent between-group findings, E_2_ was summarized narratively rather than synthesized in the network meta-analysis (**Supplementary Table S6.12**). Only two trials reported OGTT outcomes; thus, these findings were summarized descriptively (**Supplementary Table S6.13**).

### 3.6 Adverse events

Adverse events were reported in 18 studies (24, 28, 32, 38, 40, 44, 48, 52, 53, 55, 56, 60, 63, 66, 67, 69, 70, 72). Of these, six studies (32, 48, 53, 63, 66, 67) reported no adverse events. The remaining 12 studies (24, 28, 38, 40, 44, 52, 55, 56, 60, 69, 70, 72) reported adverse events, most of which were minor. Most other reported events were mild and transient, and generally resolved after cessation of the intervention or with symptomatic management. However, a small number of serious events, such as tuberculosis, were reported, and relatedness to the interventions was unclear or not assessed. In the true acupuncture group, one participant withdrew during treatment because of tuberculosis; the original report did not provide an assessment of relatedness to the intervention. Most studies reported no statistically significant difference in adverse-event incidence between the intervention and control groups. Detailed results are presented in **Table 3**.

**Table 3:** Adverse events by treatment.

Adverse events	CIAA (Cases (%))	AAT (Cases (%))	Acu (Cases (%))	AIT (Cases (%))
Dizziness			4 (2.8%)	
Subcutaneous bruising			45 (24.2%)	
Gastrointestinal reactions	2 (8%)		8 (5.6%)	
allergic skin reaction		4 (4.3%)		6 (17.6%)
Tuberculosis			1 (0.9%)	
Cholecystitis			1 (0.9%)	
Fatigue			1 (0.9%)	
Abnormal vaginal bleeding			1 (0.9%)	
Acupoint pain			7 (16.7%)	

CIAA, catgut implantation at acupoint, AAT, acupoint application therapy, Acu, acupuncture, AIT, acupoint injection therapy.

### 3.7 Small sample evaluation

**Figure 5:**
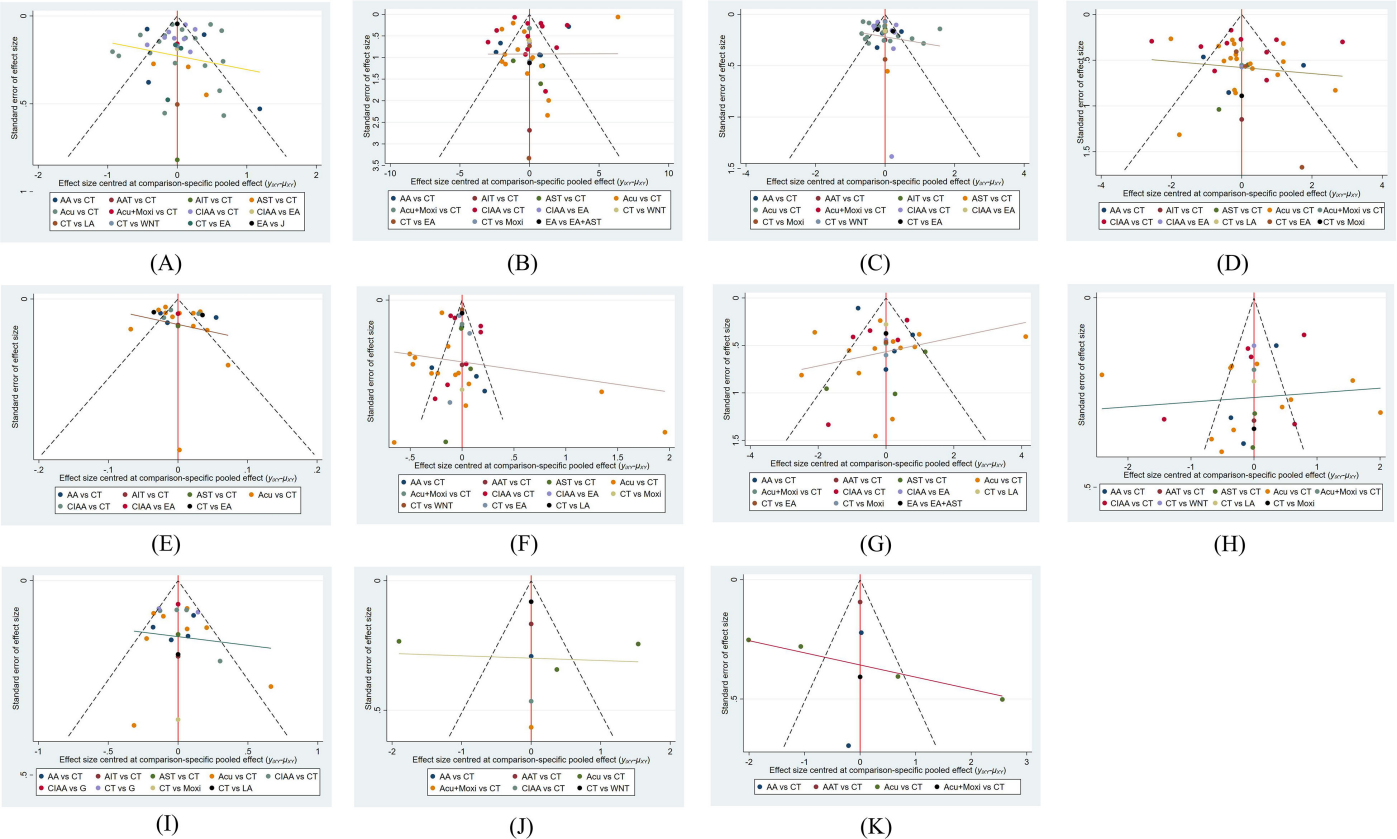
Funnel plot of all studies on different outcome measures. **(A)** homeostatic model assessment of insulin resistance (HOMA-IR); **(B)** fasting insulin (FINS); **(C)** fasting blood glucose (FBG); **(D)** body mass index (BMI); **(E)** waist-to-hip ratio (WHR); **(F)** testosterone **(T)**; **(G)** LH (luteinizing hormone); **(H)** FSH (follicle-stimulating hormone); **(I)** LH/FSH; **(J)** antral follicle count (AFC); **(K)** ovarian volume (OV). CT, conventional treatment; Acu, acupuncture; LA, laser acupuncture; AA, abdominal acupuncture; EA, electroacupuncture; CIAA, catgut implantation at acupoint; WNT, warm needle acupuncture; AST, auricular seed therapy; AAT, acupoint application therapy; Moxi, moxibustion; AIT, acupoint injection therapy.

All funnel plots show that the study points are approximately symmetrically distributed on either side of the central axis. This symmetry indicates a relatively low likelihood of publication bias in this study. These findings are illustrated in **Figure 5**.

## 4 Discussion

### 4.1 Explanation of the research results

We found that ARTs were associated with reductions in IR-related metabolic outcomes, reproductive endocrine outcomes, and ovarian morphology in women with PCOS. In this NMA of 53 studies involving 4,406 participants, ARTs were compared with conventional medications and/or placebo. Several ARTs demonstrated benefits across key outcomes, particularly reductions in FINS and AFC. Reported adverse events were generally mild; however, adverse-event reporting was limited across trials.

Based on SUCRA rankings, AIT showed the highest probability of improving IR–related markers, including HOMA-IR and FINS. LA appeared particularly beneficial for reducing T and LH and improving the LH/FSH ratio. CIAA and AA showed favorable performance for anthropometric outcomes (BMI and WHR). Overall, Acu ranked highly across multiple outcomes.

Notably, the improvement in the LH/FSH ratio in our network appears to be driven predominantly by reductions in LH rather than increases in FSH, as FSH did not show statistically significant changes across interventions (all 95% CIs crossed zero). This pattern is biologically plausible. PCOS is characterized by accelerated GnRH pulse frequency, which preferentially increases pituitary LH secretion and contributes to ovarian androgen excess; in contrast, lower GnRH pulse frequencies tend to favor FSH secretion (77, 78). Therefore, interventions that modulate hypothalamic–pituitary signaling may yield a clearer and more consistent effect on LH than on FSH. In addition, LH (and the LH/FSH ratio) may be more sensitive to short-term changes in GnRH pulsatility, whereas any small effects on FSH could be diluted by heterogeneity in hormone sampling time points and assays across trials (78).

### 4.2 Comparison with other studies

There has been a recent increase in NMA studies on PCOS, but few focus on acupuncture therapies to improve glucose metabolism in these patients. For example, WEI et al. (79) assessed acupuncture's effect on ovulation rates and outlined dosage parameters, such as the number of acupoints, treatment frequency, and duration, but did not examine glucose metabolism indicators. WANG et al.'s meta-analysis (80) addressed glucose metabolism by comparing acupuncture and metformin for IR in PCOS across 11 RCTs. Metformin was more effective for HOMA-IR, while acupuncture reduced fasting plasma glucose (FPG) more. However, this study had few trials and did not compare different acupuncture therapies. YU et al. (81) used NMA to assess ART effects on IR-related outcomes in obese PCOS, finding that Moxi combined with CIAA was most effective for FBG, FINS, and IR. This study, however, focused solely on obese patients and combined ARTs with metformin, making it difficult to assess the true effectiveness of each ART.

This study analyzed 53 articles covering all major ARTs for PCOS. It systematically assessed how these therapies improve glucose metabolism, body mass index, waist-to-hip ratio, sex hormone levels, and polycystic ovarian morphology. The results showed that different acupuncture modalities have distinct strengths with these indicators and may offer potential benefits compared with conventional treatments, while safety appeared acceptable based on reported adverse events, although adverse-event reporting was limited. Unlike traditional meta-analyses, network meta-analyses (NMAs) use direct and indirect comparisons across multiple interventions to identify the best treatments.

### 4.3 Limitations and clinical significance

This study has several limitations. Firstly, the literature selection criteria were restricted to research published in Chinese and English, excluding papers in other languages. This limitation may result in an incomplete research perspective and a lack of global representativeness. Secondly, although the search strategy primarily focused on peer-reviewed journals, non-journal literature, such as conference abstracts, was excluded due to considerations regarding data quality and reproducibility. This may also affect the completeness of the results. Furthermore, for certain acupuncture therapies, the number of studies available for analysis was limited, with only 1 or 2 meeting the criteria. This scarcity of data may introduce potential bias and restrict the generalizability of the findings. Thirdly, EA parameters were not standardized across trials and were incompletely reported in some studies (**Supplementary Table S4.1**), which may contribute to clinical heterogeneity and affect comparability. Fourthly, due to the inherent characteristics of acupuncture treatment, blinding is difficult to implement. Treatment typically relies on interaction and communication between practitioner and patient; this non-blinded status may influence patients' subjective perceptions and responses to treatment. Fifthly, conventional treatment comparators varied across trials, which may challenge the transitivity assumption and contribute to clinical heterogeneity. Lastly, adverse event reporting is crucial for assessing the safety of acupuncture. However, most original studies fail to systematically record or report relevant adverse event information. Future research should place greater emphasis on safety indicators and evaluate them. In summary, we recommend conducting subsequent large-sample, long-course, multicenter, standardized RCTs with extended follow-up periods to further validate the efficacy and safety of acupuncture treatment.

## 5 Conclusion

ARTs may be safe and effective in improving IR-related outcomes, reproductive endocrine outcomes, and polycystic ovarian morphology in patients with PCOS, suggesting a potential role as a complementary and alternative medicine (CAM) approach. However, given the limitations of the available evidence, including heterogeneity in intervention protocols and incomplete adverse-event reporting, these findings should be interpreted cautiously and warrant confirmation in well-designed, adequately powered RCTs with standardized protocols and rigorous safety reporting.

## Data Availability

The original contributions presented in the study are included in the article/**Supplementary Material**. Further inquiries can be directed to the corresponding authors.
