# Supplementary material for: Efficacy and safety of acupuncture-related therapies in improving insulin resistance, reproductive endocrine outcomes, and ovarian morphology in polycystic ovary syndrome: a systematic review and network meta-analysis
Source: Front Endocrinol (Lausanne). 2026 Feb 27;17:1748814. doi: 10.3389/fendo.2026.1748814 (PMC12983064; doi:10.3389/fendo.2026.1748814)
Supplement: Supplementary file 1 [file DataSheet1.docx]

Contents

[Supplementary TABLE S1: PRISMA guideline 2](#_Toc221190065)

[Supplementary TABLE S2: Search Strategy <inception to September 6, 2025> 5](#_Toc221190066)

[TABLE S2.1 Database: PubMed 5](#_Toc221190067)

[TABLE S2.2 Database: Web of science 6](#_Toc221190068)

[TABLE S2.3 Database: Embase 6](#_Toc221190069)

[TABLE S2.4 Database: Cochrane Library 7](#_Toc221190070)

[TABLE S2.5 Database: Chinese database 7](#_Toc221190071)

[Supplementary TABLE S3: Definitions of different therapies and conventional treatment 8](#_Toc221190072)

[Supplementary TABLE S4 Characteristics of the included studies 9](#_Toc221190073)

[Supplementary TABLE S4.1 Detailed EA parameters of included studies 11](#_Toc221190074)

[Supplementary TABLE S5 Methodological quality evaluation table 12](#_Toc221190075)

[Supplementary TABLE S6 Results of network meta-analysis 13](#_Toc221190076)

[Supplementary TABLE S6.1 Results of network meta-analysis of HOMA-IR 13](#_Toc221190077)

[Supplementary TABLE S6.2 Results of network meta-analysis of FINS 14](#_Toc221190078)

[Supplementary TABLE S6.3 Results of network meta-analysis of FBG 15](#_Toc221190079)

[Supplementary TABLE S6.4 Results of network meta-analysis of BMI 16](#_Toc221190080)

[Supplementary TABLE S6.5 Results of network meta-analysis of WHR 17](#_Toc221190081)

[Supplementary TABLE S6.6 Results of network meta-analysis of T 17](#_Toc221190082)

[Supplementary TABLE S6.7 Results of network meta-analysis of LH 18](#_Toc221190083)

[Supplementary TABLE S6.8 Results of network meta-analysis of FSH 18](#_Toc221190084)

[Supplementary TABLE S6.9 Results of network meta-analysis of LH/FSH 19](#_Toc221190085)

[Supplementary TABLE S6.10 Results of network meta-analysis of AFC 20](#_Toc221190086)

[Supplementary TABLE S6.11 Results of network meta-analysis of OV 20](#_Toc221190087)

[Supplementary TABLE S6.12 E_2_ outcomes (exploratory) 21](#_Toc221190088)

[Supplementary TABLE S6.13 OGTT outcomes (exploratory) 22](#_Toc221190089)

[Supplementary TABLE S7 Node-Splitting Results for Local Inconsistency Test 23](#_Toc221190090)

[Supplementary TABLE S7.1 Node-Splitting Results of HOMA-IR 23](#_Toc221190091)

[Supplementary TABLE S7.2 Node-Splitting Results of FINS 23](#_Toc221190092)

[Supplementary TABLE S7.3 Node-Splitting Results of FBG 24](#_Toc221190093)

[Supplementary TABLE S7.4 Node-Splitting Results of BMI 24](#_Toc221190094)

[Supplementary TABLE S7.5 Node-Splitting Results of WHR 25](#_Toc221190095)

[Supplementary TABLE S7.6 Node-Splitting Results of T 25](#_Toc221190096)

[Supplementary TABLE S7.7 Node-Splitting Results of LH 26](#_Toc221190097)

[Supplementary TABLE S7.8 Node-Splitting Results of FSH 26](#_Toc221190098)

[Supplementary TABLE S7.9 Node-Splitting Results of LH/FSH 27](#_Toc221190099)

[Supplementary TABLE S7.10 Node-Splitting Results of AFC 27](#_Toc221190100)

[Supplementary TABLE S7.11 Node-Splitting Results of OV 28](#_Toc221190101)

[Supplementary TABLE S8 Loop Inconsistency Factor Test Results in Network Meta-Analysis 28](#_Toc221190102)

[Supplementary FIGURE: Forest Plot of Network Meta-Analysis with Consistency Test 30](#_Toc221190103)

[**Reference** 33](#_Toc221190104)

# Supplementary TABLE S1: PRISMA guideline

| **Section and Topic** | **Item #** | **Checklist item** | **Location where item is reported** |
| --- | --- | --- | --- |
| **TITLE** | | |  |
| Title | 1 | Identify the report as a systematic review. | Title |
| **ABSTRACT** | | |  |
| Abstract | 2 | See the PRISMA 2020 for Abstracts checklist. | Abstract |
| **INTRODUCTION** | | |  |
| Rationale | 3 | Describe the rationale for the review in the context of existing knowledge. | Introduction |
| Objectives | 4 | Provide an explicit statement of the objective(s) or question(s) the review addresses. | Introduction |
| **METHODS** | | |  |
| Eligibility criteria | 5 | Specify the inclusion and exclusion criteria for the review and how studies were grouped for the syntheses. | Methods |
| Information sources | 6 | Specify all databases, registers, websites, organisations, reference lists and other sources searched or consulted to identify studies. Specify the date when each source was last searched or consulted. | Methods |
| Search strategy | 7 | Present the full search strategies for all databases, registers and websites, including any filters and limits used. | Supplementary Table S2 |
| Selection process | 8 | Specify the methods used to decide whether a study met the inclusion criteria of the review, including how many reviewers screened each record and each report retrieved, whether they worked independently, and if applicable, details of automation tools used in the process. | Figure 1 |
| Data collection process | 9 | Specify the methods used to collect data from reports, including how many reviewers collected data from each report, whether they worked independently, any processes for obtaining or confirming data from study investigators, and if applicable, details of automation tools used in the process. | Methods |
| Data items | 10a | List and define all outcomes for which data were sought. Specify whether all results that were compatible with each outcome domain in each study were sought (e.g. for all measures, time points, analyses), and if not, the methods used to decide which results to collect. | Methods |
|  | 10b | List and define all other variables for which data were sought (e.g. participant and intervention characteristics, funding sources). Describe any assumptions made about any missing or unclear information. | Methods |
| Study risk of bias assessment | 11 | Specify the methods used to assess risk of bias in the included studies, including details of the tool(s) used, how many reviewers assessed each study and whether they worked independently, and if applicable, details of automation tools used in the process. | Methods |
| Effect measures | 12 | Specify for each outcome the effect measure(s) (e.g. risk ratio, mean difference) used in the synthesis or presentation of results. | Methods |
| Synthesis methods | 13a | Describe the processes used to decide which studies were eligible for each synthesis (e.g. tabulating the study intervention characteristics and comparing against the planned groups for each synthesis (item #5)). | Supplementary Table S3 |
|  | 13b | Describe any methods required to prepare the data for presentation or synthesis, such as handling of missing summary statistics, or data conversions. | Methods |
|  | 13c | Describe any methods used to tabulate or visually display results of individual studies and syntheses. | Methods |
|  | 13d | Describe any methods used to synthesize results and provide a rationale for the choice(s). If meta-analysis was performed, describe the model(s), method(s) to identify the presence and extent of statistical heterogeneity, and software package(s) used. | Methods |
|  | 13e | Describe any methods used to explore possible causes of heterogeneity among study results (e.g. subgroup analysis, meta-regression). | Methods |
|  | 13f | Describe any sensitivity analyses conducted to assess robustness of the synthesized results. | Methods |
| Reporting bias assessment | 14 | Describe any methods used to assess risk of bias due to missing results in a synthesis (arising from reporting biases). | Methods |
| Certainty assessment | 15 | Describe any methods used to assess certainty (or confidence) in the body of evidence for an outcome. | Methods |
| **RESULTS** | | |  |
| Study selection | 16a | Describe the results of the search and selection process, from the number of records identified in the search to the number of studies included in the review, ideally using a flow diagram. | Figure 1 |
|  | 16b | Cite studies that might appear to meet the inclusion criteria, but which were excluded, and explain why they were excluded. | Figure 1 |
| Study characteristics | 17 | Cite each included study and present its characteristics. | Supplementary Table S3 |
| Risk of bias in studies | 18 | Present assessments of risk of bias for each included study. | Supplementary Table S5, Figure 3 |
| Results of individual studies | 19 | For all outcomes, present, for each study: (a) summary statistics for each group (where appropriate) and (b) an effect estimate and its precision (e.g. confidence/credible interval), ideally using structured tables or plots. | Results |
| Results of syntheses | 20a | For each synthesis, briefly summarise the characteristics and risk of bias among contributing studies. | Results |
|  | 20b | Present results of all statistical syntheses conducted. If meta-analysis was done, present for each the summary estimate and its precision (e.g. confidence/credible interval) and measures of statistical heterogeneity. If comparing groups, describe the direction of the effect. | Table 2, Figure 4-5 |
|  | 20c | Present results of all investigations of possible causes of heterogeneity among study results. | Results |
|  | 20d | Present results of all sensitivity analyses conducted to assess the robustness of the synthesized results. | - |
| Reporting biases | 21 | Present assessments of risk of bias due to missing results (arising from reporting biases) for each synthesis assessed. | Results |
| Certainty of evidence | 22 | Present assessments of certainty (or confidence) in the body of evidence for each outcome assessed. | Results |
| **DISCUSSION** | | |  |
| Discussion | 23a | Provide a general interpretation of the results in the context of other evidence. | Discussion |
|  | 23b | Discuss any limitations of the evidence included in the review. | Discussion |
|  | 23c | Discuss any limitations of the review processes used. | Discussion |
|  | 23d | Discuss implications of the results for practice, policy, and future research. | Discussion |
| **OTHER INFORMATION** | | |  |
| Registration and protocol | 24a | Provide registration information for the review, including register name and registration number, or state that the review was not registered. | Methods |
|  | 24b | Indicate where the review protocol can be accessed, or state that a protocol was not prepared. | Methods |
|  | 24c | Describe and explain any amendments to information provided at registration or in the protocol. | Methods |
| Support | 25 | Describe sources of financial or non-financial support for the review, and the role of the funders or sponsors in the review. | Discussion |
| Competing interests | 26 | Declare any competing interests of review authors. | Discussion |
| Availability of data, code and other materials | 27 | Report which of the following are publicly available and where they can be found: template data collection forms; data extracted from included studies; data used for all analyses; analytic code; any other materials used in the review. | Discussion |

# Supplementary TABLE S2: Search Strategy <inception to September 6, 2025>

# TABLE S2.1 Database: PubMed

| ID | Search Terms | Results |
| --- | --- | --- |
| #1 | Polycystic Ovary Syndrome[MeSH Terms] | 20358 |
| #2 | (((((((((((((Ovary Syndrome, Polycystic[Title/Abstract])) OR (Syndrome, Polycystic Ovary[Title/Abstract])) OR (Polycystic Ovarian Syndrome[Title/Abstract])) OR (Ovarian Syndrome, Polycystic[Title/Abstract])) OR (Sclerocystic Ovarian Degeneration[Title/Abstract])) OR (Ovarian Degeneration, Sclerocystic[Title/Abstract])) OR (Sclerocystic Ovary Syndrome[Title/Abstract])) OR (Stein-Leventhal Syndrome[Title/Abstract])) OR (Stein Leventhal Syndrome[Title/Abstract])) OR (Syndrome, Stein-Leventhal[Title/Abstract])) OR (Sclerocystic Ovaries[Title/Abstract])) OR (Ovary, Sclerocystic[Title/Abstract])) OR (Sclerocystic Ovary[Title/Abstract]) | 5553 |
| #3 | #1 OR #2 | 22329 |
| #4 | (((((Acupuncture[MeSH Terms]) OR (Acupuncture Therapy[MeSH Terms])) OR (Acupuncture, Ear[MeSH Terms])) OR (Acupuncture Points[MeSH Terms])) OR (Electroacupuncture[MeSH Terms])) OR (Moxibustion[MeSH Terms]) | 33226 |
| #5 | ((((((((((((((((((Pharmacopuncture[Title/Abstract]) OR (Acupuncture Treatment*[Title/Abstract])) OR (Treatment, Acupuncture[Title/Abstract])) OR (Therapy, Acupuncture[Title/Abstract])) OR (Pharmacoacupuncture Treatment[Title/Abstract])) OR (Treatment, Pharmacoacupuncture[Title/Abstract])) OR (Pharmacoacupuncture Therapy[Title/Abstract])) OR (Therapy, Pharmacoacupuncture[Title/Abstract])) OR (Acupotomy[Title/Abstract])) OR (Acupotomies[Title/Abstract])) OR (Acupunctures, Ear[Title/Abstract])) OR (Ear Acupunctures[Title/Abstract])) OR (Acupuncture*, Auricular[Title/Abstract])) OR (Auricular Acupuncture*[Title/Abstract])) OR (Ear Acupuncture[Title/Abstract])) OR (Acupuncture Point[Title/Abstract])) OR (Point*, Acupuncture[Title/Abstract])) OR (Acupoint*[Title/Abstract])) OR (Electroacupuncture[Title/Abstract]) | 19455 |
| #6 | #4 OR #5 | 37823 |
| #7 | #3 AND #6 | 225 |

# TABLE S2.2 Database: Web of science

| ID | Search Terms | Results |
| --- | --- | --- |
| #1 | TS=(Polycystic Ovary Syndrome OR Ovary Syndrome, Polycystic OR Syndrome, Polycystic Ovary OR Polycystic Ovarian Syndrome OR Ovarian Syndrome, Polycystic OR Sclerocystic Ovarian Degeneration OR Ovarian Degeneration, Sclerocystic OR Sclerocystic Ovary Syndrome OR Stein-Leventhal Syndrome OR Stein Leventhal Syndrome OR Syndrome, Stein-Leventhal OR Sclerocystic Ovaries OR Ovary, Sclerocystic OR Sclerocystic Ovary) | 24355 |
| #2 | TS=(Acupuncture OR Acupuncture Therapy OR Acupuncture, Ear OR Acupuncture Points OR Electroacupuncture OR Moxibustion OR Pharmacopuncture OR Acupuncture Treatment* OR Treatment, Acupuncture OR Therapy, Acupuncture OR Pharmacoacupuncture Treatment OR Treatment, Pharmacoacupuncture OR Pharmacoacupuncture Therapy OR Therapy, Pharmacoacupuncture OR Acupotomy OR Acupotomies OR Acupunctures, Ear OR Ear Acupunctures OR Acupuncture*, Auricular OR Auricular Acupuncture* OR Ear Acupuncture OR Acupuncture Point OR Point*, Acupuncture OR Acupoint* OR Electroacupuncture) | 25468 |
| #3 | #1 AND #2 | 267 |

# TABLE S2.3 Database: Embase

| ID | Search Terms | Results |
| --- | --- | --- |
| #1 | 'ovary polycystic disease'/exp OR 'ovary polycystic disease'/exp | 45,284 |
| #2 | 'ovary syndrome, polycystic':ti,ab,kw OR 'syndrome, polycystic ovary':ti,ab,kw OR 'polycystic ovarian syndrome':ti,ab,kw OR 'ovarian syndrome, polycystic':ti,ab,kw OR 'sclerocystic ovarian degeneration':ti,ab,kw OR 'ovarian degeneration, sclerocystic':ti,ab,kw OR 'sclerocystic ovary syndrome':ti,ab,kw OR 'stein-leventhal syndrome':ti,ab,kw OR 'stein leventhal syndrome':ti,ab,kw OR 'syndrome, stein-leventhal':ti,ab,kw OR 'sclerocystic ovaries':ti,ab,kw OR 'ovary, sclerocystic':ti,ab,kw OR 'sclerocystic ovary':ti,ab,kw | 8,793 |
| #3 | 'acupuncture'/exp OR 'acupuncture therapy'/exp OR 'acupuncture, ear'/exp OR 'acupuncture points'/exp OR 'electroacupuncture'/exp OR 'moxibustion'/exp | 70,767 |
| #4 | 'pharmacopuncture':ti,ab,kw OR 'acupuncture treatment*':ti,ab,kw OR 'treatment, acupuncture':ti,ab,kw OR 'therapy, acupuncture':ti,ab,kw OR 'pharmacoacupuncture treatment':ti,ab,kw OR 'treatment, pharmacoacupuncture':ti,ab,kw OR 'pharmacoacupuncture therapy':ti,ab,kw OR 'therapy, pharmacoacupuncture':ti,ab,kw OR 'acupotomy':ti,ab,kw OR 'acupotomies':ti,ab,kw OR 'acupunctures, ear':ti,ab,kw OR 'ear acupunctures':ti,ab,kw OR 'acupuncture*, auricular':ti,ab,kw OR 'auricular acupuncture*':ti,ab,kw OR 'ear acupuncture':ti,ab,kw OR 'acupuncture point':ti,ab,kw OR 'point*, acupuncture':ti,ab,kw OR 'acupoint*':ti,ab,kw OR 'electroacupuncture':ti,ab,kw | 26,121 |
| #5 | #1 OR #2 | 46,737 |
| #6 | #3 OR #4 | 71,726 |
| #7 | #5 AND #6 | 516 |

# TABLE S2.4 Database: Cochrane Library

| ID | Search Terms | Results |
| --- | --- | --- |
| #1 | MeSH descriptor: [Polycystic Ovary Syndrome] explode all trees | 2182 |
| #2 | (Ovary Syndrome, Polycystic OR Syndrome, Polycystic Ovary OR Polycystic Ovarian Syndrome OR Ovarian Syndrome, Polycystic OR Sclerocystic Ovarian Degeneration OR Ovarian Degeneration, Sclerocystic OR Sclerocystic Ovary Syndrome OR Stein-Leventhal Syndrome OR Stein Leventhal Syndrome OR Syndrome, Stein-Leventhal OR Sclerocystic Ovaries OR Ovary, Sclerocystic OR Sclerocystic Ovary):ti, ab,kw | 5322 |
| #3 | #1 OR #2 | 5322 |
| #4 | MeSH descriptor: [Acupuncture] explode all trees | 216 |
| #5 | MeSH descriptor: [Acupuncture Therapy] explode all trees | 7245 |
| #6 | MeSH descriptor: [Acupuncture, Ear] explode all trees | 276 |
| #7 | MeSH descriptor: [Acupuncture Points] explode all trees | 2875 |
| #8 | MeSH descriptor: [Electroacupuncture] explode all trees | 1202 |
| #9 | MeSH descriptor: [Moxibustion] explode all trees | 698 |
| #10 | (Pharmacopuncture OR Acupuncture Treatment OR Treatment, Acupuncture OR Therapy, Acupuncture OR Pharmacoacupuncture Treatment OR Treatment, Pharmacoacupuncture OR Pharmacoacupuncture Therapy OR Therapy, Pharmacoacupuncture OR Acupotomy OR Acupotomies OR Acupunctures, Ear OR Ear Acupunctures OR Acupuncture, Auricular OR Auricular Acupuncture OR Ear Acupuncture OR Acupuncture Point OR Point, Acupuncture OR Acupoint* OR Electroacupuncture):ti,ab,kw | 23174 |
| #11 | #4 OR #5 OR #6 OR #7 OR #8 OR #9 OR #10 | 24455 |
| #12 | #3 AND #11 | 216 |

# TABLE S2.5 Database: Chinese database

| Database | Search Terms | Results |
| --- | --- | --- |
| CNKI | (SU =('Acu' + '电针' +'温针疗法' +'头针' +'腹针' + '腕踝针' + '火针' + '芒针' + '灸法' + '温和灸' + '药灸' + '督灸' + '悬灸' + '热敏灸' + '麦粒灸' + '电灸' +'隔药饼灸' +'隔姜灸' + '隔附子饼灸' + '隔棉灸' + '艾条灸' + '艾炷灸' + '雷火灸' + '间接灸' + '直接灸'+ '穴位贴敷' + '穴位埋线' + '水针' + '耳针') * '多囊卵巢综合征') OR (TKA = ('针' + '灸' + '穴') * ('多囊卵巢综合征' + '多囊卵巢综合症')) | 1566 |
| Wanfang | (主题:(Acu or 电针 or 温针疗法 or 头针 or 腹针 or 腕踝针 or 火针 or 芒针 or 灸法 or 温和灸 or 药灸 or 督灸 or 悬灸 or 热敏灸 or 麦粒灸 or 电灸 or 隔药饼灸 or 隔姜灸 or 隔附子饼灸 or 隔棉灸 or 艾条灸 or 艾炷灸 or 雷火灸 or 间接灸 or 直接灸'+ '穴位贴敷 or 穴位埋线 or 水针 or 耳针) or 题名或关键词:(针 or 灸 or 穴)) and ( 主题:(多囊卵巢综合征) or 题名或关键词:(多囊卵巢综合征 or 多囊卵巢综合症)) | 2028 |
| VIP | ((U=Acu OR U=电针 OR U=温针疗法 OR U=头针 OR U=腹针 OR U=腕踝针 OR U=火针 OR U=芒针 OR U=灸法 OR U=温和灸 OR U=药灸 OR U=督灸 OR U=热敏灸 OR U=麦粒灸 OR U=隔药饼灸 OR U=隔姜灸 OR U=隔附子饼灸 OR U=隔棉灸 OR U=艾条灸 OR U=艾炷灸 OR U=雷火灸 OR U=间接灸 OR U=直接灸 OR U=穴 OR U=穴位贴敷 OR U=穴位埋线 OR U=水针 OR U=耳针) OR (M=针 OR M=灸 OR M=穴)) AND (M=多囊卵巢综合征 OR M=多囊卵巢综合症) | 989 |
| CBM | (("针"[常用字段:智能]) OR ("穴"[常用字段:智能]) OR ("灸"[常用字段:智能])) AND ("多囊卵巢综合征"[常用字段:智能]) | 1904 |

# Supplementary TABLE S3: Definitions of different therapies and conventional treatment

| abbreviation | Full name | Definitions |
| --- | --- | --- |
| CT | conventional treatment | The conventional treatment in this study is conventional hormone treatment. |
| Acu | acupuncture | Acupuncture, also known as needling or acupuncture therapy, is a traditional Chinese medical treatment method that involves stimulating specific points on the body with needles to regulate qi and blood, balance yin and yang, and promote self-healing of the body. |
| LA | laser acupuncture | Laser Acupuncture is a novel acupuncture technique that focuses or spreads low-intensity laser beams onto acupoints, integrating traditional acupuncture theory with modern laser technology. Compared to traditional acupuncture, laser acupuncture offers advantages such as eliminating needle-prick pain, fainting, broken needles, and infection risks. |
| AA | abdominal acupuncture | Abdominal Acupuncture is an acupuncture treatment based on the meridian theory of Traditional Chinese Medicine (TCM), which regulates the body's visceral functions and meridian qi and blood through stimulation of specific acupuncture points in the abdomen, thereby treating systemic diseases. |
| EA | electroacupuncture | Electroacupuncture is a traditional Chinese medical treatment method that involves stimulating specific acupuncture points on the body using special electroacupuncture needles to regulate qi and blood, promote blood circulation, and alleviate pain, among other therapeutic purposes. |
| CIAA | catgut implantation at acupoint | Catgut implantation at acupoint is a traditional Chinese medical treatment method that involves inserting one or more special threads under the skin at specific acupoints on the body, providing continuous stimulation to regulate qi and blood, promote tendon relaxation, and treat diseases. |
| WNT | warm needle acupuncture | Warm Needle Acupuncture is a therapeutic method combining needle insertion with moxibustion, also known as needle-handle moxibustion. The technique involves inserting a filiform needle until de qi is achieved, then wrapping moxa wool onto the needle handle or attaching a 1-2 cm segment of moxa stick. Once ignited, the heat is transmitted through the needle shaft into the acupoint, delivering the dual effects of both acupuncture and moxibustion. |
| AST | auricular seed therapy | Auricular Seed Therapy is a traditional Chinese medicine treatment method, which belongs to the ear acupuncture therapy. It is through the specific points in the ear on the paste pressure small beans (usually WangBuLiuXing seeds or other small particles of material), the use of its continuous stimulation of the ear acupuncture points, to achieve the regulation of the human body's internal organs function, treatment of disease and health care purposes. |
| AAT | acupoint application therapy | Acupoint Application Therapy is a traditional Chinese medicine external treatment method, by applying drugs or other substances directly to specific acupoints, utilizing the medicinal effect of the drugs and the stimulating effect of the acupoints to achieve the purpose of preventing and treating diseases. |
| Moxi | moxibustion | Moxibustion, also known as moxa therapy or ai zhi, is a traditional Chinese medical treatment method. It involves burning moxa (dried mugwort) at specific points on the body or placing it directly on the skin to apply heat and stimulate acupuncture points, regulating qi and blood circulation, relaxing tendons, and boosting the immune system. It includes main types such as direct moxibustion, indirect moxibustion, and moxa stick moxibustion. |
| AIT | acupoint injection therapy | Acupoint injection therapy involves administering sterile solutions into acupoint locations to enhance therapeutic effects. This approach integrates traditional acupuncture point theory with modern injection techniques, utilizing substances such as plant extracts, vitamins, and homeopathic remedies. |

# Supplementary TABLE S4 Characteristics of the included studies

| Author and Year | Average age | | Number of patients | | Intervention | | Treatment duration | Outcomes |
| --- | --- | --- | --- | --- | --- | --- | --- | --- |
|  | T | C | T | C | T | C |  |  |
| Wu (2024)(1) | 26.95±3.8 | 27.28±2.74 | 39 | 42 | Clo +Acu | Clo | 3months | ①②③⑦⑧⑨ |
| El-Shamy (2018)(2) | 20.2±0.3 | 19.8±0.8 | 11 | 10 | LA | SA | 3months | ①④⑥⑦⑧⑨ |
| Zheng (2013)(3) | 26.5±3.0 | 24.9±4.9 | 43 | 43 | AA | Met | 6months | ①④⑤⑦⑧⑨ |
| Muharam (2022)(4) | 27.91±4.09 | 28.14±3.21 | 22 | 22 | EA+Met | SA+Met | 1months | ②③④ |
| Wen (2022)(5) | 27.0±4.44 | 27.0±3.7 | 114 | 114 | Acu+Placebo | SA+Placebo | 4months | ① |
| Cai (2016)(6) | 29.5±2.3 | | 25; 25 | 25 | EA | Met | 3months | ①②③④⑤⑥⑦⑨ |
| Chai (2021)(7) | 28.6±3.4 | 28.5±3.5 | 50 | 50 | Acu+TCM | TCM | 3months | ①②③⑥⑧⑩⑪ |
| Dun (2018)(8) | 16.9±1.58 | 17±1.82 | 30 | 30 | Met+AA | Met | 3months | ①②③④⑤⑥⑦⑧⑨ |
| Gu (2019)(9) | 26.95±4.54 | 28.56±3.98 | 39 | 39 | Acu | SA | 16weeks | ①②③④⑤ |
| Ha (2019)(10) | 31.14±4.21 | 31.20±4.23 | 40 | 40 | TCM+Acu | TCM | 3months | ②③④⑥⑦⑧ |
| Jiang (2015)(11) | 23. 22±3.54 | 23. 04±3. 44 | 41 | 43 | TCM+CIAA | TCM | 3months | ①②③④⑤ |
| Jiang (2017)(12) | 25.03±4.29 | 24.78±4.35 | 45 | 45 | TCM+WNT | TCM | 3months | ①②③⑥⑧⑩ |
| Jiang (2014)(13) | 25. 23±1. 45 | 26. 12 ± 1. 02 | 48 | 48 | Met+CIAA | Met | 3months | ①②③④⑥⑦⑧⑨ |
| Jiang (2023)(14) | 30.68±4.58 | 30.13±4.89 | 42 | 42 | Met+Acu | Met | 3months | ①②③④⑤ |
| Lai (2012)(15) | 26.72±2.65 | 26.46±2.72 | 60 | 60 | AA | Met | 4months | ①②③④⑤⑥⑦⑨ |
| Li (2015)(16) | 36.44±3.11 | 36．89±4．19 | 36 | 36 | EA+AST | EA | 1months | ①②⑦ |
| Liu (2024)(17) | 28.36±3.41 | 28.14±3.29 | 93 | 93 | TCM+AAT | TCM | 9months | ①③⑥⑦⑧⑩⑪ |
| Liu (2017)(18) | 29.12±6.35 | 28.67±5.67 | 44 | 44 | TCM+Acu | TCM | 3months | ②③⑥⑦⑧ |
| Mao (2021)(19) | NA | | 54 | 54 | Met+ EE&CPA +Acu | Met+EE&CPA | 3months | ①④⑤ |
| Peng (2017)(20) | 28.58±3.82 | 28.68±3.33 | 50 | 50 | Acu | SA | 3months | ④⑤⑥⑨ |
| Quan (2021)(21) | 23.80±6.98 | 22.97±6.21 | 30 | 30 | Met+Acu | Met | 3months | ①②③④⑤⑥⑦⑧⑨⑩⑪ |
| Su (2013)(22) | 25.69±4.97 | 28.15±5.23 | 40 | 40 | TCM+Acu | TCM | 3months | ①②③④⑥⑦⑧⑨ |
| Song (2024)(23) | 26.12±2.42 | 25.96±2.84 | 50 | 50 | EE&CPA +CIAA | EE&CPA | 3months | ①②③④⑥⑦⑧⑨ |
| Su (2025)(24) | 29.88±2.91 | 29.39±3.16 | 42 | 38 | Met+Acu | Met | 3months | ①②③④⑤⑥⑦⑧⑨ |
| Tao (2008)(25) | NA | | 22 | 20 | TCM+CIAA | TCM | 3months | ②③④ |
| Wang (2021)(26) | 28.4±5.01 | 28.52±5.55 | 30 | 29 | CC+Acu+Moxi | CC | 3months | ①②③④⑥⑦⑧⑩⑪ |
| Wang (2016)(27) | 29.43±4.35 | | 49 | 40 | TCM+Acu | TCM | 3months | ①②③④ |
| Wang (2025)(28) | 30.38±5.01 | 29.80±4.68 | 40 | 40 | Met+CIAA | Met | 3months | ①②③④⑥⑦⑧ |
| Wang (2023)(29) | 28.20±4.77 | 28.00±5.12 | 25 | 25 | Met+TCM+CIAA | Met + TCM | 3months | ①②③⑥⑨ |
| Wang (2022)(30) | 28.3±4.19 | 28.42±3.79 | 20 | 20 | TCM+Acu | TCM | 2months | ①④⑤ |
| Wang (2015)(31) | 23-44 | | 35 | 35 | Moxi | EE&CPA | 3months | ②③④⑥⑦⑨⑧ |
| Wu (2025)(32) | 26.80±4.78 | 26.95±4.92 | 34 | 36 | LG+AIT | LG | 3months | ①②③④⑤⑨ |
| Xu R H (2024)(33) | 26.85±5.76 | 26.54±5.56 | 30 | 30 | LG+Met+Acu | LG+ Met | 3months | ①②③④⑤⑥⑦⑨ |
| Xu Y (2024)(34) | 35.62±4.68 | 34.98±5.01 | 74 | 74 | EE&CPA +CIAA | EE&CPA | 3months | ①②⑥⑦⑧⑩ |
| Yang (2021)(35) | 26.72±1.08 | | 36 | 36 | TCM+CIAA | TCM | 3months | ②④ |
| Yang (2022)(36) | 28.19±3.87 | 27.17±4.42 | 47 | 47 | TCM+Acu | TCM +SA | 3months | ①②③⑥⑦⑧ |
| Yao (2018)(37) | 27.8±4.8 | 28.2±4.5 | 50 | 50 | Met+EA | Met | 6months | ①④⑤⑥⑨ |
| Ye (2018)(38) | 25.6±0.6 | | 57 | 57 | TCM+ CIAA | TCM | 3months | ②④ |
| Yin (2016)(39) | 27.54±2.03 | 27.56±2.01 | 30 | 30 | Met+AA | Met | 3months | ①②③ |
| Zhang (2025)(40) | 27.46±4.24 | 28.49±3.40 | 28 | 28 | TCM+AST | TCM | 3months | ①②④⑤⑥⑦ |
| Zhang (2017)(41) | 28.3±4.2 | 27.9±4.3 | 64 | 64 | Met+Acu | Met | 3months | ①②③④⑥⑦⑨⑩⑪ |
| Zhao (2022)(42) | 30.50±4.35 | 29.5±4.25 | 26 | 26 | TCM+CIAA | TCM | 3months | ②③④⑤⑥⑦⑧ |
| Zhu (2025)(43) | 28.70 ±3.71 | 28.00 ±4.16 | 30 | 30 | TCM+AST | TCM | 24weeks | ①②③⑥⑦⑧ |
| Li (2018)(44) | 14-19 | 15-19 | 30 | 30 | AST | LG | 3months | ①②③④⑥⑦⑧⑨ |
| Wang (2020)(45) | 26.43±3.52 | 26.23±3.48 | 30 | 30 | Met+Acu | Met | 3months | ③④⑤⑦⑧ |
| Lai (2010)(46) | 24.9±4.9 | 26.5±3.0 | 43 | 43 | AA | Met | 6months | ①②③④⑤⑥⑦⑧⑨⑪ |
| Huang (2025)(47) | 28.79±5.58 | 27.35±3.64 | 40 | 40 | LG+Acu | LG | 3months | ①②③④⑥⑦⑧ |
| Chen (2021)(48) | 26.85±2.55 | 26.83±2.56 | 40 | 40 | AA | Clo | 3months | ②⑥⑦⑧⑨⑩⑪ |
| Li (2024)(49) | 27.15±5.21 | 26.34±4.26 | 33 | 32 | LG+Acu | LG+ Met | 3months | ①②③④⑤⑥⑦⑧ |
| Ma (2020)(50) | 26±2 | 25±1 | 42 | 42 | Met+CIAA | Met | 3months | ①②④ |
| Zhao (2007)(51) | 27.1±3.5 | | 30 | 30 | Met+Acu | Met | 3months | ①③④⑤⑥⑨ |
| Zhang (2022)(52) | 28.52±2.64 | 29.26±3.27 | 60 | 60 | CC +Acu | CC | 3months | ①④⑤⑥⑦⑪ |
| Yu (2019)(53) | 30±6 | 31±6 | 31 | 30 | EA | Met | 3months | ①⑥ |

Note: T, trial group; C, control group; CT, conventional treatment; Acu, acupuncture; LA, laser acupuncture; AA, abdominal acupuncture; EA, electroacupuncture; CIAA, catgut implantation at acupoint; WNT, warm needle acupuncture; AST, auricular seed therapy; AAT, acupoint application therapy; Moxi, moxibustion; AIT, acupoint injection therapy; NA, not available; Clo, Clomiphene; Met, Metformin; EE&CPA, Ethinylestradiol and Cyproterone Acetate; CC, Clomiphene Citrate; TCM, traditional Chinese medicine; ①homeostatic model assessment of insulin resistance (HOMA-IR); ②fasting insulin (FINS); ③fasting blood glucose (FBG); ④body mass index (BMI); ⑤waist-to-hip ratio (WHR); ⑥testosterone (T); ⑦LH (luteinizing hormone); ⑧FSH (follicle-stimulating hormone); ⑨LH/FSH; ⑩antral follicle count (AFC); ⑪ ovarian volume (OV)

# Supplementary TABLE S4.1 Detailed EA parameters of included studies

| Articles | Acupoints | Frequency (Hz) | Intensity (mA) | waveform | Session duration (minutes | Sessions per week (times) | Total course (months) |
| --- | --- | --- | --- | --- | --- | --- | --- |
| Muharam (2022)(4) | Zhongji (CV3), Guanyuan (CV4), Qihai (CV6), Tianshu (ST25), Shuidao (ST28), Zusanli (ST36), Chengshan (BL57), and Sanyinjiao (SP6) | 2 | to patient comfort | continuous wave | 30 | 3 | 1 |
| Cai (2016)(6) | Zhongwan (CV12), Guanyuan (CV4), Tianshu (ST25), Daimai (GB26), Zusanli (ST36) and Sanyinjiao (SP6) | - | to the maximum tolerable intensity | continuous wave | 30 | 3 | 3 |
| Li (2015)(16) | Guanyuan (CV4), Zhongji (CV3), Sanyinjiao (SP6) and Zigong (EX-CA1) | 3~4 | 2~3 | intermittent pulse wave | 30 | 1 | 1 |
| Yao (2018)(37) | Tianshu (ST25), Zigong (EX-CA1), Zhongwan (CV12), and Guanyuan (CV4) | 20 | to the maximum tolerable intensity | continuous wave | 30 | 3 | 6 |
| Yu (2019)(53) | Yishu (EX-B3), Pishu (BL20), Ciliao (BL32) and Shenshu (BL23) | 2 | to the maximum tolerable intensity | continuous wave | 30 | 3 | 1 |

Note: electroacupuncture, EA

# Supplementary TABLE S5 Methodological quality evaluation table

| Articles | Random sequence generation | Allocation concealment | Blinding of participants and personnel | Blinding of outcome assessment | Incomplete outcome data | Selective outcome reporting | Other |
| --- | --- | --- | --- | --- | --- | --- | --- |
| Wu (2024)(1) | low | low | Unclear | Unclear | Low | Low | Unclear |
| El-Shamy (2018)(2) | low | low | low | Unclear | Low | Low | Unclear |
| Zheng (2013)(3) | low | low | Unclear | Unclear | Low | Low | Unclear |
| Muharam (2022)(4) | low | low | low | Unclear | Low | Low | Unclear |
| Wen (2022)(5) | low | low | low | Unclear | Low | Low | Unclear |
| Cai (2016)(6) | Low | Unclear | Unclear | Unclear | Low | Low | Unclear |
| Chai (2021)(7) | low | Unclear | Unclear | Unclear | Low | Low | Unclear |
| Dun (2018)(8) | High | Unclear | Unclear | Unclear | Low | Low | Unclear |
| Gu (2019)(9) | low | Unclear | Unclear | Unclear | high | Low | Unclear |
| Ha (2019)(10) | unclear | Unclear | Unclear | Unclear | Low | Low | Unclear |
| Jiang (2015)(11) | low | Unclear | Unclear | Unclear | high | Low | Unclear |
| Jiang (2017)(12) | low | Unclear | Unclear | Unclear | Low | Low | Unclear |
| Jiang (2014)(13) | Unclear | Unclear | Unclear | Unclear | Low | Low | Unclear |
| Jiang (2023)(14) | low | Unclear | Unclear | Unclear | Low | Low | Unclear |
| Lai (2012)(15) | Unclear | Unclear | Unclear | Unclear | Low | Low | Unclear |
| Li (2015)(16) | low | Unclear | Unclear | Unclear | Low | Low | Unclear |
| Liu (2024)(17) | low | Unclear | Unclear | Unclear | Low | Low | Unclear |
| Liu (2017)(18) | low | Unclear | Unclear | Unclear | Low | Low | Unclear |
| Mao (2021)(19) | low | Unclear | Unclear | Unclear | Low | Low | Unclear |
| Peng (2017)(20) | Unclear | Unclear | Unclear | Unclear | Low | Low | Unclear |
| Quan (2021)(21) | Unclear | Unclear | Unclear | Unclear | Low | Low | Unclear |
| Su (2013)(22) | low | Unclear | Unclear | Unclear | Low | Low | Unclear |
| Song (2024)(23) | low | Unclear | Unclear | Unclear | Low | Low | Unclear |
| Su (2025)(24) | low | Unclear | Unclear | Unclear | Low | Low | Unclear |
| Tao (2008)(25) | Unclear | Unclear | Unclear | Unclear | Low | Low | Unclear |
| Wang (2021)(26) | Unclear | Unclear | Unclear | Unclear | high | Low | Unclear |
| Wang (2016)(27) | Unclear | Unclear | Unclear | Unclear | Low | Low | Unclear |
| Wang (2025)(28) | low | Unclear | Unclear | Unclear | high | Low | Unclear |
| Wang (2023)(29) | low | Unclear | Unclear | Unclear | Low | Low | Unclear |
| Wang (2022)(30) | low | Unclear | Unclear | Unclear | high | Low | Unclear |
| Wang (2015)(31) | low | Unclear | Unclear | Unclear | Low | Low | Unclear |
| Wu (2025)(32) | low | Unclear | Unclear | Unclear | Low | Low | Unclear |
| Xu R H (2024)(33) | low | Unclear | Unclear | Unclear | Low | Low | Unclear |
| Xu Y (2024)(34) | low | Unclear | Unclear | Unclear | Low | Low | Unclear |
| Yang (2021)(35) | Unclear | Unclear | Unclear | Unclear | Low | Low | Unclear |
| Yang (2022)(36) | low | Unclear | Unclear | Unclear | Low | Low | Unclear |
| Yao (2018)(37) | low | Unclear | Unclear | Unclear | Low | Low | Unclear |
| Ye (2018)(38) | Unclear | Unclear | Unclear | Unclear | Low | Low | Unclear |
| Yin (2016)(39) | low | Unclear | Unclear | Unclear | Low | Low | Unclear |
| Zhang (2025)(40) | low | Unclear | Unclear | Unclear | Low | Low | Unclear |
| Zhang (2017)(41) | low | Unclear | Unclear | Unclear | Low | Low | Unclear |
| Zhao (2022)(42) | Unclear | Unclear | Unclear | Unclear | Low | Low | Unclear |
| Zhu (2025)(43) | low | Unclear | Unclear | Unclear | high | Low | Unclear |
| Li (2018)(44) | Unclear | Unclear | Unclear | Unclear | Low | Low | Unclear |
| Wang (2020)(45) | low | Unclear | Unclear | Unclear | Low | Low | Unclear |
| Lai (2010)(46) | low | Unclear | Unclear | Unclear | Low | Low | Unclear |
| Huang (2025)(47) | low | Unclear | Unclear | Unclear | Low | Low | Unclear |
| Chen (2021)(48) | low | Unclear | Unclear | Unclear | Low | Low | Unclear |
| Li (2024)(49) | low | Unclear | Unclear | Unclear | Low | Low | Unclear |
| Ma (2020)(50) | low | Unclear | Unclear | Unclear | Low | Low | Unclear |
| Zhao (2007)(51) | Unclear | Unclear | Unclear | Unclear | Low | Low | Unclear |
| Zhang (2022)(52) | Unclear | Unclear | Unclear | Unclear | Low | Low | Unclear |
| Yu (2019)(53) | low | Unclear | Unclear | Unclear | high | Low | Unclear |

# Supplementary TABLE S6 Results of network meta-analysis

# Supplementary TABLE S6.1 Results of network meta-analysis of HOMA-IR

| Interventions | CT | EA | LA | AST | EA+AST | WNT | CIAA | AA | AAT | Acu | Acu+Moxi | AIT |
| --- | --- | --- | --- | --- | --- | --- | --- | --- | --- | --- | --- | --- |
| AIT | 2.20 (0.44,3.96) | 1.97 (0.15,3.80) | 2.00 (-0.14,4.14) | 1.86 (0.02,3.71) | 1.84 (-0.12,3.80) | 1.77 (-0.16,3.70) | 1.75 (-0.03,3.53) | 1.71 (-0.09,3.52) | 1.50 (-0.40,3.40) | 1.57 (-0.20,3.34) | 1.14 (-0.78,3.06) | 0 |
| Acu+Moxi | 1.06 (0.28,1.84) | 0.83 (-0.09,1.75) | 0.86 (-0.59,2.31) | 0.72 (-0.24,1.68) | 0.70 (-0.47,1.87) | 0.63 (-0.48,1.74) | 0.61 (-0.21,1.44) | 0.57 (-0.31,1.45) | 0.36 (-0.71,1.43) | 0.43 (-0.38,1.24) | 0 |  |
| Acu | 0.63 (0.44,0.83) | 0.40 (-0.12,0.92) | 0.43 (-0.81,1.67) | 0.29 (-0.30,0.88) | 0.27 (-0.62,1.16) | 0.20 (-0.62,1.02) | 0.18 (-0.15,0.51) | 0.14 (-0.30,0.59) | -0.07 (-0.82,0.68) | 0 |  |  |
| AAT | 0.70 (-0.02,1.42) | 0.47 (-0.40,1.34) | 0.50 (-0.92,1.92) | 0.36 (-0.55,1.28) | 0.34 (-0.79,1.47) | 0.27 (-0.80,1.34) | 0.25 (-0.52,1.02) | 0.21 (-0.62,1.04) | 0 |  |  |  |
| AA | 0.49 (0.09,0.89) | 0.26 (-0.37,0.89) | 0.29 (-1.00,1.57) | 0.15 (-0.54,0.84) | 0.13 (-0.83,1.09) | 0.06 (-0.83,0.95) | 0.04 (-0.44,0.52) | 0 |  |  |  |  |
| CIAA | 0.45 (0.18,0.71) | 0.22 (-0.30,0.74) | 0.25 (-1.00,1.50) | 0.11 (-0.51,0.73) | 0.09 (-0.80,0.98) | 0.02 (-0.82,0.85) | 0 |  |  |  |  |  |
| WNT | 0.43 (-0.36,1.22) | 0.20 (-0.73,1.13) | 0.23 (-1.23,1.69) | 0.09 (-0.88,1.06) | 0.07 (-1.10,1.25) | 0 |  |  |  |  |  |  |
| EA+AST | 0.36 (-0.51,1.23) | 0.13 (-0.59,0.85) | 0.16 (-1.34,1.66) | 0.02 (-1.01,1.05) | 0 |  |  |  |  |  |  |  |
| AST | 0.34 (-0.22,0.90) | 0.11 (-0.63,0.85) | 0.14 (-1.20,1.48) | 0 |  |  |  |  |  |  |  |  |
| LA | 0.20 (-1.02,1.42) | -0.03 (-1.34,1.28) | 0 |  |  |  |  |  |  |  |  |  |
| EA | 0.23 (-0.25,0.71) | 0 |  |  |  |  |  |  |  |  |  |  |
| CT | 0 |  |  |  |  |  |  |  |  |  |  |  |

Note: CT, conventional treatment; Acu, acupuncture; LA, laser acupuncture; AA, abdominal acupuncture; EA, electroacupuncture; CIAA, catgut implantation at acupoint; WNT, warm needle acupuncture; AST, auricular seed therapy; AAT, acupoint application therapy; Moxi, moxibustion; AIT, acupoint injection therapy; homeostatic model assessment of insulin resistance (HOMA-IR)

# Supplementary TABLE S6.2 Results of network meta-analysis of FINS

| Interventions | CT | EA | Acu+Moxi | AST | AA | EA+AST | Moxi | Acu | WNT | CIAA | AIT |
| --- | --- | --- | --- | --- | --- | --- | --- | --- | --- | --- | --- |
| AIT | 7.30 (0.83,13.77) | 6.49 (-0.72,13.70) | 6.27 (-1.24,13.78) | 6.04 (-0.94,13.01) | 4.97 (-1.75,11.68) | 4.47 (-3.96,12.90) | 4.19 (-3.59,11.97) | 4.33 (-2.23,10.89) | 3.89 (-3.69,11.47) | 4.19 (-2.38,10.76) | 0 |
| CIAA | 3.11 (1.97,4.25) | 2.30 (-0.92,5.52) | 2.08 (-1.90,6.06) | 1.85 (-1.00,4.70) | 0.78 (-1.36,2.92) | 0.28 (-5.14,5.70) | 0.00 (-4.47,4.47) | 0.14 (-1.44,1.72) | -0.30 (-4.41,3.81) | 0 |  |
| WNT | 3.41 (-0.54,7.36) | 2.60 (-2.48,7.67) | 2.38 (-3.11,7.87) | 2.15 (-2.59,6.88) | 1.08 (-3.27,5.42) | 0.58 (-6.11,7.27) | 0.30 (-5.55,6.15) | 0.44 (-3.65,4.54) | 0 |  |  |
| Acu | 2.97 (1.87,4.06) | 2.16 (-1.22,5.53) | 1.94 (-2.03,5.90) | 1.71 (-1.13,4.54) | 0.63 (-1.48,2.75) | 0.14 (-5.38,5.65) | -0.14 (-4.60,4.32) | 0 |  |  |  |
| Moxi | 3.11 (-1.21,7.43) | 2.30 (-3.08,7.67) | 2.08 (-3.68,7.84) | 1.85 (-3.20,6.90) | 0.78 (-3.91,5.46) | 0.28 (-6.64,7.20) | 0 |  |  |  |  |
| EA+AST | 2.83 (-2.57,8.23) | 2.02 (-2.34,6.38) | 1.80 (-4.81,8.41) | 1.57 (-4.43,7.57) | 0.50 (-5.20,6.20) | 0 |  |  |  |  |  |
| AA | 2.33 (0.52,4.14) | 1.52 (-2.15,5.19) | 1.30 (-2.92,5.52) | 1.07 (-2.11,4.25) | 0 |  |  |  |  |  |  |
| AST | 1.26 (-1.35,3.87) | 0.45 (-3.67,4.58) | 0.23 (-4.39,4.85) | 0 |  |  |  |  |  |  |  |
| Acu+Moxi | 1.03 (-2.78,4.84) | 0.22 (-4.75,5.19) | 0 |  |  |  |  |  |  |  |  |
| EA | 0.81 (-2.38,4.00) | 0 |  |  |  |  |  |  |  |  |  |
| CT | 0 |  |  |  |  |  |  |  |  |  |  |

Note: CT, conventional treatment; Acu, acupuncture; AA, abdominal acupuncture; EA, electroacupuncture; CIAA, catgut implantation at acupoint; WNT, warm needle acupuncture; AST, auricular seed therapy; Moxi, moxibustion; AIT, acupoint injection therapy; fasting insulin (FINS)

# Supplementary TABLE S6.3 Results of network meta-analysis of FBG

| Interventions | CT | AIT | AST | CIAA | Acu+Moxi | EA | AA | Moxi | WNT | AAT | Acu |
| --- | --- | --- | --- | --- | --- | --- | --- | --- | --- | --- | --- |
| Acu | 0.56 (0.31,0.81) | 0.62 (-0.24,1.48) | 0.65 (-0.67,1.97) | 0.50 (-0.23,1.22) | 0.50 (-0.51,1.51) | 0.34 (-0.24,0.92) | 0.33 (-0.13,0.78) | 0.19 (-0.85,1.23) | 0.10 (-0.91,1.11) | -0.07 (-1.18,1.04) | 0 |
| AAT | 0.63 (-0.46,1.72) | 0.69 (-0.67,2.05) | 0.72 (-0.97,2.41) | 0.57 (-0.71,1.84) | 0.57 (-0.89,2.03) | 0.41 (-0.80,1.62) | 0.39 (-0.75,1.54) | 0.26 (-1.22,1.74) | 0.17 (-1.29,1.63) | 0 |  |
| WNT | 0.46 (-0.52,1.44) | 0.52 (-0.76,1.79) | 0.55 (-1.07,2.17) | 0.40 (-0.79,1.58) | 0.40 (-0.98,1.78) | 0.24 (-0.87,1.35) | 0.22 (-0.82,1.27) | 0.09 (-1.31,1.49) | 0 |  |  |
| Moxi | 0.37 (-0.64,1.38) | 0.43 (-0.87,1.73) | 0.46 (-1.18,2.10) | 0.31 (-0.91,1.52) | 0.31 (-1.09,1.71) | 0.15 (-0.99,1.29) | 0.13 (-0.94,1.21) | 0 |  |  |  |
| AA | 0.24 (-0.14,0.61) | 0.29 (-0.61,1.20) | 0.33 (-1.02,1.67) | 0.17 (-0.55,0.89) | 0.18 (-0.87,1.22) | 0.01 (-0.63,0.66) | 0 |  |  |  |  |
| EA | 0.22 (-0.30,0.75) | 0.28 (-0.69,1.25) | 0.31 (-1.09,1.71) | 0.16 (-0.70,1.01) | 0.16 (-0.95,1.27) | 0 |  |  |  |  |  |
| Acu+Moxi | 0.06 (-0.92,1.04) | 0.12 (-1.16,1.39) | 0.15 (-1.47,1.77) | -0.00 (-1.19,1.18) | 0 |  |  |  |  |  |  |
| CIAA | 0.06 (-0.61,0.74) | 0.12 (-0.94,1.18) | 0.15 (-1.31,1.62) | 0 |  |  |  |  |  |  |  |
| AST | -0.09 (-1.39,1.21) | -0.03 (-1.56,1.50) | 0 |  |  |  |  |  |  |  |  |
| AIT | -0.06 (-0.88,0.76) | 0 |  |  |  |  |  |  |  |  |  |
| CT | 0 |  |  |  |  |  |  |  |  |  |  |

Note: CT, conventional treatment; Acu, acupuncture; AA, abdominal acupuncture; EA, electroacupuncture; CIAA, catgut implantation at acupoint; WNT, warm needle acupuncture; AST, auricular seed therapy; AAT, acupoint application therapy; Moxi, moxibustion; AIT, acupoint injection therapy; fasting blood glucose (FBG)

# Supplementary TABLE S6.4 Results of network meta-analysis of BMI

| Interventions | AA | CT | CIAA | Moxi | AIT | AST | LA | EA | Acu | Acu+Moxi |
| --- | --- | --- | --- | --- | --- | --- | --- | --- | --- | --- |
| Acu+Moxi | 6.40 (3.06,9.74) | 5.80 (3.38,8.22) | 5.40 (2.57,8.23) | 4.77 (0.82,8.72) | 4.56 (1.49,7.63) | 4.24 (0.55,7.93) | 3.88 (1.38,6.37) | 3.52 (0.79,6.26) | 3.51 (0.98,6.04) | 0 |
| Acu | 2.89 (0.49,5.29) | 2.29 (1.57,3.01) | 1.89 (0.34,3.44) | 1.26 (-1.94,4.47) | 1.05 (-0.96,3.06) | 0.73 (-2.14,3.60) | 0.37 (-0.56,1.30) | 0.02 (-1.45,1.48) | 0 |  |
| EA | 2.88 (0.25,5.50) | 2.28 (1.00,3.55) | 1.87 (-0.06,3.81) | 1.25 (-2.13,4.62) | 1.04 (-1.23,3.30) | 0.72 (-2.34,3.77) | 0.35 (-1.05,1.76) | 0 |  |  |
| LA | 2.52 (0.16,4.89) | 1.92 (1.33,2.51) | 1.52 (-0.05,3.09) | 0.89 (-2.29,4.07) | 0.68 (-1.29,2.65) | 0.36 (-2.48,3.21) | 0 |  |  |  |
| AST | 2.16 (-1.44,5.76) | 1.56 (-1.22,4.34) | 1.16 (-1.98,4.30) | 0.53 (-3.65,4.71) | 0.32 (-3.03,3.68) | 0 |  |  |  |  |
| AIT | 1.84 (-1.12,4.80) | 1.24 (-0.64,3.12) | 0.84 (-1.54,3.21) | 0.21 (-3.43,3.85) | 0 |  |  |  |  |  |
| Moxi | 1.63 (-2.24,5.50) | 1.03 (-2.09,4.15) | 0.63 (-2.82,4.07) | 0 |  |  |  |  |  |  |
| CIAA | 1.00 (-1.71,3.72) | 0.40 (-1.06,1.86) | 0 |  |  |  |  |  |  |  |
| CT | 0.60 (-1.69,2.89) | 0 |  |  |  |  |  |  |  |  |
| AA | 0 |  |  |  |  |  |  |  |  |  |

Note: CT, conventional treatment; Acu, acupuncture; LA, laser acupuncture; AA, abdominal acupuncture; EA, electroacupuncture; CIAA, catgut implantation at acupoint; AST, auricular seed therapy; Moxi, moxibustion; AIT, acupoint injection therapy; body mass index (BMI)

# Supplementary TABLE S6.5 Results of network meta-analysis of WHR

| Interventions | CT | AST | AIT | CIAA | AA | Acu | EA |
| --- | --- | --- | --- | --- | --- | --- | --- |
| EA | 0.06 (0.02,0.09) | 0.05 (-0.03,0.12) | 0.04 (-0.04,0.11) | 0.02 (-0.03,0.08) | 0.02 (-0.02,0.05) | 0.01 (-0.04,0.06) | 0 |
| Acu | 0.05 (0.01,0.08) | 0.04 (-0.04,0.11) | 0.03 (-0.05,0.10) | 0.01 (-0.03,0.06) | 0.01 (-0.03,0.05) | 0 |  |
| AA | 0.04 (0.02,0.06) | 0.03 (-0.04,0.10) | 0.02 (-0.05,0.09) | 0.01 (-0.04,0.05) | 0 |  |  |
| CIAA | 0.03 (-0.01,0.07) | 0.02 (-0.06,0.10) | 0.01 (-0.07,0.09) | 0 |  |  |  |
| AIT | 0.02 (-0.05,0.09) | 0.01 (-0.09,0.11) | 0 |  |  |  |  |
| AST | 0.01 (-0.06,0.08) | 0 |  |  |  |  |  |
| CT | 0 |  |  |  |  |  |  |

Note: CT, conventional treatment; Acu, acupuncture; AA, abdominal acupuncture; EA, electroacupuncture; CIAA, catgut implantation at acupoint; AST, auricular seed therapy; AIT, acupoint injection therapy; waist-to-hip ratio (WHR);

# Supplementary TABLE S6.6 Results of network meta-analysis of T

| Interventions | CT | Moxi | Acu+Moxi | Acu | EA | AST | AA | WNT | CIAA | AAT | LA |
| --- | --- | --- | --- | --- | --- | --- | --- | --- | --- | --- | --- |
| LA | 0.59 (0.33,0.85) | 0.57 (-0.05,1.18) | 0.59 (-0.38,1.56) | 0.50 (-0.08,1.09) | 0.49 (-0.47,1.46) | 0.48 (-0.53,1.49) | 0.41 (-0.03,0.85) | 0.38 (-0.58,1.35) | 0.30 (-0.25,0.84) | -0.01 (-1.00,0.98) | 0 |
| AAT | 0.60 (-0.35,1.55) | 0.57 (-0.54,1.68) | 0.60 (-0.74,1.94) | 0.51 (-0.58,1.60) | 0.50 (-0.83,1.83) | 0.49 (-0.88,1.86) | 0.41 (-0.60,1.43) | 0.39 (-0.94,1.72) | 0.31 (-0.76,1.37) | 0 |  |
| CIAA | 0.29 (-0.18,0.77) | 0.27 (-0.47,1.01) | 0.29 (-0.75,1.34) | 0.20 (-0.51,0.91) | 0.19 (-0.85,1.24) | 0.18 (-0.90,1.27) | 0.11 (-0.49,0.70) | 0.08 (-0.96,1.13) | 0 |  |  |
| WNT | 0.21 (-0.72,1.14) | 0.18 (-0.91,1.27) | 0.21 (-1.11,1.53) | 0.12 (-0.95,1.19) | 0.11 (-1.21,1.43) | 0.10 (-1.25,1.45) | 0.02 (-0.97,1.02) | 0 |  |  |  |
| AA | 0.19 (-0.17,0.54) | 0.16 (-0.51,0.82) | 0.19 (-0.81,1.18) | 0.09 (-0.50,0.68) | 0.09 (-0.91,1.08) | 0.08 (-0.96,1.11) | 0 |  |  |  |  |
| AST | 0.11 (-0.87,1.09) | 0.08 (-1.04,1.21) | 0.11 (-1.24,1.46) | 0.02 (-1.09,1.13) | 0.01 (-1.34,1.36) | 0 |  |  |  |  |  |
| EA | 0.10 (-0.83,1.03) | 0.07 (-1.02,1.16) | 0.10 (-1.22,1.42) | 0.01 (-1.06,1.08) | 0 |  |  |  |  |  |  |
| Acu | 0.09 (-0.43,0.62) | 0.06 (-0.71,0.83) | 0.09 (-0.98,1.16) | 0 |  |  |  |  |  |  |  |
| Acu+Moxi | -0.00 (-0.93,0.93) | -0.03 (-1.12,1.06) | 0 |  |  |  |  |  |  |  |  |
| Moxi | 0.03 (-0.54,0.59) | 0 |  |  |  |  |  |  |  |  |  |
| CT | 0 |  |  |  |  |  |  |  |  |  |  |

Note: CT, conventional treatment; Acu, acupuncture; LA, laser acupuncture; AA, abdominal acupuncture; EA, electroacupuncture; CIAA, catgut implantation at acupoint; WNT, warm needle acupuncture; AST, auricular seed therapy; AAT, acupoint application therapy; Moxi, moxibustion; testosterone (T)

# Supplementary TABLE S6.7 Results of network meta-analysis of LH

| Interventions | CT | AST | Moxi | AA | AAT | Acu+Moxi | EA | EA+AST | CIAA | LA | Acu |
| --- | --- | --- | --- | --- | --- | --- | --- | --- | --- | --- | --- |
| Acu | 2.89 (2.12,3.66) | 3.09 (0.25,5.94) | 2.23 (0.35,4.12) | 1.97 (0.54,3.40) | 1.80 (-0.89,4.49) | 1.50 (-1.25,4.26) | 1.49 (-0.98,3.97) | 1.18 (-2.39,4.76) | 0.71 (-0.62,2.05) | -0.11 (-2.75,2.54) | 0 |
| LA | 3.00 (0.47,5.53) | 3.20 (-0.53,6.93) | 2.34 (-0.72,5.40) | 2.07 (-0.73,4.88) | 1.91 (-1.70,5.52) | 1.61 (-2.05,5.27) | 1.60 (-1.86,5.05) | 1.29 (-3.02,5.60) | 0.82 (-1.94,3.58) | 0 |  |
| CIAA | 2.18 (1.09,3.27) | 2.38 (-0.57,5.33) | 1.52 (-0.51,3.56) | 1.25 (-0.37,2.88) | 1.09 (-1.71,3.89) | 0.79 (-2.07,3.65) | 0.78 (-1.57,3.13) | 0.47 (-3.02,3.96) | 0 |  |  |
| EA+AST | 1.71 (-1.78,5.20) | 1.91 (-2.53,6.35) | 1.05 (-2.84,4.94) | 0.78 (-2.91,4.47) | 0.62 (-3.72,4.96) | 0.32 (-4.06,4.70) | 0.31 (-2.27,2.89) | 0 |  |  |  |
| EA | 1.40 (-0.95,3.75) | 1.60 (-2.01,5.21) | 0.74 (-2.17,3.65) | 0.47 (-2.17,3.11) | 0.31 (-3.18,3.80) | 0.01 (-3.53,3.55) | 0 |  |  |  |  |
| Acu+Moxi | 1.39 (-1.26,4.04) | 1.59 (-2.22,5.40) | 0.73 (-2.42,3.89) | 0.46 (-2.44,3.37) | 0.30 (-3.39,3.99) | 0 |  |  |  |  |  |
| AAT | 1.09 (-1.49,3.67) | 1.29 (-2.47,5.05) | 0.43 (-2.67,3.53) | 0.16 (-2.68,3.01) | 0 |  |  |  |  |  |  |
| AA | 0.93 (-0.28,2.13) | 1.13 (-1.87,4.12) | 0.27 (-1.83,2.37) | 0 |  |  |  |  |  |  |  |
| Moxi | 0.66 (-1.06,2.38) | 0.86 (-2.38,4.09) | 0 |  |  |  |  |  |  |  |  |
| AST | -0.20 (-2.94,2.54) | 0 |  |  |  |  |  |  |  |  |  |
| CT | 0 |  |  |  |  |  |  |  |  |  |  |

Note: CT, conventional treatment; Acu, acupuncture; LA, laser acupuncture; AA, abdominal acupuncture; EA, electroacupuncture; CIAA, catgut implantation at acupoint; AST, auricular seed therapy; AAT, acupoint application therapy; Moxi, moxibustion; LH (luteinizing hormone);

# Supplementary TABLE S6.8 Results of network meta-analysis of FSH

| Interventions | CIAA | AA | AST | WNT | CT | Acu+Moxi | LA | Moxi | AAT | Acu |
| --- | --- | --- | --- | --- | --- | --- | --- | --- | --- | --- |
| Acu | 0.94 (-1.03,2.91) | 0.63 (-0.51,1.78) | 0.65 (-1.42,2.72) | 0.47 (-0.57,1.51) | 0.42 (-0.16,1.01) | 0.46 (-1.53,2.45) | 0.42 (-1.58,2.43) | 0.14 (-1.39,1.66) | -0.27 (-2.32,1.79) | 0 |
| AAT | 1.21 (-1.52,3.94) | 0.90 (-1.31,3.10) | 0.92 (-1.88,3.72) | 0.74 (-1.41,2.89) | 0.69 (-1.28,2.66) | 0.73 (-2.01,3.47) | 0.69 (-2.06,3.44) | 0.40 (-2.02,2.83) | 0 |  |
| Moxi | 0.81 (-1.55,3.16) | 0.49 (-1.22,2.21) | 0.52 (-1.92,2.95) | 0.33 (-1.32,1.98) | 0.29 (-1.12,1.69) | 0.33 (-2.04,2.69) | 0.29 (-2.09,2.66) | 0 |  |  |
| LA | 0.52 (-2.17,3.21) | 0.21 (-1.95,2.36) | 0.23 (-2.53,2.99) | 0.05 (-2.05,2.15) | -0.00 (-1.92,1.92) | 0.04 (-2.66,2.74) | 0 |  |  |  |
| Acu+Moxi | 0.48 (-2.20,3.16) | 0.17 (-1.97,2.31) | 0.19 (-2.56,2.94) | 0.01 (-2.08,2.09) | -0.04 (-1.94,1.86) | 0 |  |  |  |  |
| CT | 0.52 (-1.36,2.40) | 0.21 (-0.78,1.19) | 0.23 (-1.76,2.22) | 0.05 (-0.81,0.90) | 0 |  |  |  |  |  |
| WNT | 0.47 (-1.60,2.54) | 0.16 (-1.14,1.47) | 0.18 (-1.98,2.35) | 0 |  |  |  |  |  |  |
| AST | 0.29 (-2.45,3.03) | -0.02 (-2.24,2.19) | 0 |  |  |  |  |  |  |  |
| AA | 0.31 (-1.81,2.44) | 0 |  |  |  |  |  |  |  |  |
| CIAA | 0 |  |  |  |  |  |  |  |  |  |

Note: CT, conventional treatment; Acu, acupuncture; LA, laser acupuncture; AA, abdominal acupuncture; CIAA, catgut implantation at acupoint; WNT, warm needle acupuncture; AST, auricular seed therapy; AAT, acupoint application therapy; Moxi, moxibustion; FSH (follicle-stimulating hormone);

# Supplementary TABLE S6.9 Results of network meta-analysis of LH/FSH

| Interventions | CT | Moxi | AST | AA | EA | AIT | CIAA | Acu | LA |
| --- | --- | --- | --- | --- | --- | --- | --- | --- | --- |
| LA | 0.70 (0.26,1.14) | 0.77 (0.14,1.40) | 0.66 (-0.20,1.52) | 0.56 (0.10,1.03) | 0.51 (0.04,0.99) | 0.36 (-0.21,0.93) | 0.38 (-0.09,0.84) | 0.27 (-0.19,0.72) | 0 |
| Acu | 0.43 (0.31,0.55) | 0.50 (0.04,0.97) | 0.39 (-0.36,1.14) | 0.29 (0.10,0.49) | 0.24 (0.02,0.47) | 0.09 (-0.29,0.47) | 0.11 (-0.08,0.30) | 0 |  |
| CIAA | 0.32 (0.17,0.47) | 0.39 (-0.08,0.86) | 0.28 (-0.47,1.04) | 0.18 (-0.03,0.40) | 0.14 (-0.07,0.34) | -0.02 (-0.41,0.37) | 0 |  |  |
| AIT | 0.34 (-0.02,0.70) | 0.41 (-0.16,0.98) | 0.30 (-0.52,1.12) | 0.20 (-0.19,0.59) | 0.15 (-0.25,0.56) | 0 |  |  |  |
| EA | 0.19 (0.00,0.37) | 0.26 (-0.23,0.74) | 0.15 (-0.61,0.91) | 0.05 (-0.19,0.29) | 0 |  |  |  |  |
| AA | 0.14 (-0.02,0.29) | 0.21 (-0.27,0.68) | 0.10 (-0.66,0.85) | 0 |  |  |  |  |  |
| AST | 0.04 (-0.70,0.78) | 0.11 (-0.75,0.97) | 0 |  |  |  |  |  |  |
| Moxi | -0.07 (-0.52,0.38) | 0 |  |  |  |  |  |  |  |
| CT | 0 |  |  |  |  |  |  |  |  |

Note: CT, conventional treatment; Acu, acupuncture; LA, laser acupuncture; AA, abdominal acupuncture; EA, electroacupuncture; CIAA, catgut implantation at acupoint; AST, auricular seed therapy; Moxi, moxibustion; AIT, acupoint injection therapy; LH (luteinizing hormone); FSH (follicle-stimulating hormone);

# Supplementary TABLE S6.10 Results of network meta-analysis of AFC

| Interventions | Acu+Moxi | CT | AA | AAT | CIAA | Acu | WNT |
| --- | --- | --- | --- | --- | --- | --- | --- |
| WNT | 4.56 (-0.29,9.41) | 4.08 (0.63,7.53) | 3.44 (-1.54,8.42) | 2.38 (-2.56,7.32) | 1.89 (-2.97,6.75) | 1.02 (-2.97,5.01) | 0 |
| Acu | 3.54 (-0.41,7.49) | 3.06 (1.07,5.05) | 2.42 (-1.68,6.52) | 1.36 (-2.69,5.41) | 0.87 (-3.09,4.83) | 0 |  |
| CIAA | 2.67 (-2.16,7.50) | 2.19 (-1.23,5.61) | 1.55 (-3.40,6.50) | 0.49 (-4.42,5.40) | 0 |  |  |
| AAT | 2.18 (-2.73,7.09) | 1.70 (-1.83,5.23) | 1.06 (-3.97,6.09) | 0 |  |  |  |
| AA | 1.12 (-3.83,6.07) | 0.64 (-2.94,4.22) | 0 |  |  |  |  |
| CT | 0.48 (-2.93,3.89) | 0 |  |  |  |  |  |
| Acu+Moxi | 0 |  |  |  |  |  |  |

Note: CT, conventional treatment; Acu, acupuncture; AA, abdominal acupuncture; CIAA, catgut implantation at acupoint; WNT, warm needle acupuncture; AAT, acupoint application therapy; Moxi, moxibustion; antral follicle count (AFC);

# Supplementary TABLE S6.11 Results of network meta-analysis of OV

| Interventions | CT | AA | AAT | Acu+Moxi | Acu |
| --- | --- | --- | --- | --- | --- |
| Acu | 2.38 (0.67,4.08) | 2.19 (-1.64,6.02) | 1.35 (-1.64,4.35) | 0.69 (-3.06,4.44) | 0 |
| Acu+Moxi | 1.69 (-1.65,5.03) | 1.50 (-3.29,6.29) | 0.67 (-3.48,4.81) | 0 |  |
| AAT | 1.02 (-1.44,3.48) | 0.83 (-3.39,5.05) | 0 |  |  |
| AA | 0.19 (-3.24,3.62) | 0 |  |  |  |
| CT | 0 |  |  |  |  |

Note: CT, conventional treatment; Acu, acupuncture; AA, abdominal acupuncture; AAT, acupoint application therapy; Moxi, moxibustion; ovarian volume (OV)

# Supplementary TABLE S6.12 E_2_ outcomes (exploratory)

| Articles | n | Treatment | Sampling time point (cycle day) | E2 value (after treatment)  Mean ± SD/ Median (IQR) | Units | Between-group conclusion |
| --- | --- | --- | --- | --- | --- | --- |
| Chai (2021)(7) | 50 | CT | Not reported | 40.52±10.23 | pg/mL | Higher in Acu vs CT |
|  | 50 | Acu |  | 46.37±11.86 |  |  |
| Jiang (2014)(13) | 48 | CT | Not reported | 334.49±246.8 | pmol/L | No statistically significant between-group difference |
|  | 48 | CIAA |  | 255.4±96.11 |  |  |
| Peng (2017)(20) | 50 | CT | Not reported | 61.67±16.35 | pg/mL | No statistically significant between-group difference |
|  | 50 | Acu |  | 71.88±16.82 |  |  |
| Su (2025)(24) | 38 | CT | Day 3–5 of menstruation or withdrawal bleeding | 49.3±9.88 | pg/mL | Higher in Acu vs CT |
|  | 42 | Acu |  | 56.15±10.17 |  |  |
| Yang (2022)(36) | 47 | CT | Day 2–3 of menstruation | 42.16±7.94 | pg/mL | No statistically significant between-group difference |
|  | 47 | Acu |  | 41.09±8.44 |  |  |
| Zhu (2025)(43) | 29 | CT | Day 2–4 of menstruation or withdrawal bleeding | 40.5±12.27 | pg/mL | No statistically significant between-group difference |
|  | 25 | AST |  | 34.07±13.16 |  |  |
| Wang (2020)(45) | 30 | CT | Not reported | 162.08±32.21 | ng/L | Higher in Acu vs CT |
|  | 30 | Acu |  | 225.99±15.04 |  |  |
| Li (2024)(49) | 31 | CT | Day 2 of menstruation or withdrawal bleeding | 64.3±16.5 | pmol/L | Lower in Acu vs CT |
|  | 31 | Acu |  | 53.5±19.6 |  |  |
| Wu (2024)(1) | 39 | CT | Not reported | 57.31(48.85,65.77) | pg/mL | No statistically significant between-group difference |
|  | 42 | Acu |  | 62.43(53.82,71.05) |  |  |

Note: CT, conventional treatment; Acu, acupuncture; CIAA, catgut implantation at acupoint; AST, auricular seed therapy; Units were reported as in the original trials and were not harmonized.

# Supplementary TABLE S6.13 OGTT outcomes (exploratory)

| Articles | n | Treatment | Sampling time point (cycle day) | 2-h glucose/ mmol/L | 2-h insulin/ mU/L | Between-group conclusion (2-h) |
| --- | --- | --- | --- | --- | --- | --- |
| Gu (2019)(9) | 38 | CT | Not reported | 5.18±1.99 | 49.10±31.99 | No statistically significant between-group difference for either 2-h glucose or 2-h insulin. |
|  | 38 | Acu |  | 5.61±3.06 | 50.61±43.02 |  |
| Wu (2025)(32) | 36 | CT | Not reported | 7.80±2.56 | 119.86±97.28 | No statistically significant between-group difference for either 2-h glucose or 2-h insulin. |
|  | 34 | AIT |  | 7.17±2.01 | 118.95±69.60 |  |

Note: CT, conventional treatment; Acu, acupuncture; AIT, acupoint injection therapy; OGTT, Oral glucose tolerance test; OGTT values are end-of-treatment values as reported in the original trials.

# Supplementary TABLE S7 Node-Splitting Results for Local Inconsistency Test

# Supplementary TABLE S7.1 Node-Splitting Results of HOMA-IR


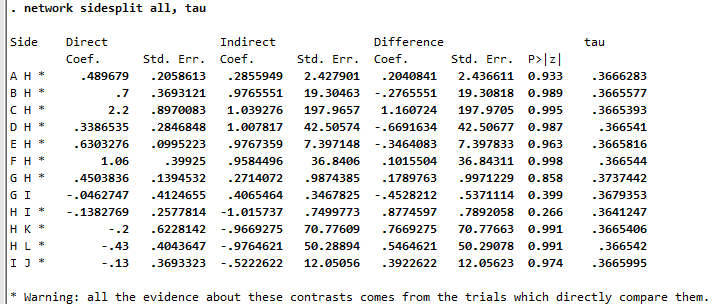


# Supplementary TABLE S7.2 Node-Splitting Results of FINS


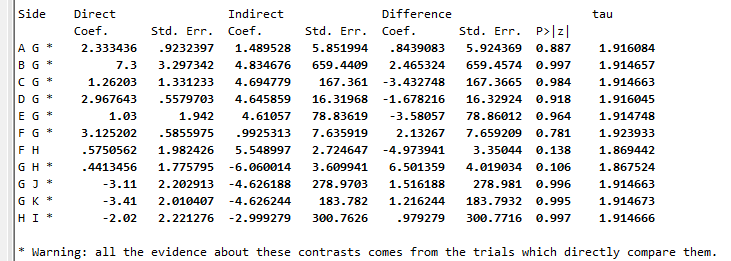


# Supplementary TABLE S7.3 Node-Splitting Results of FBG


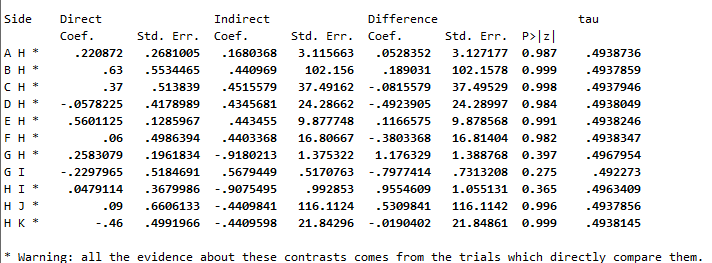


# Supplementary TABLE S7.4 Node-Splitting Results of BMI


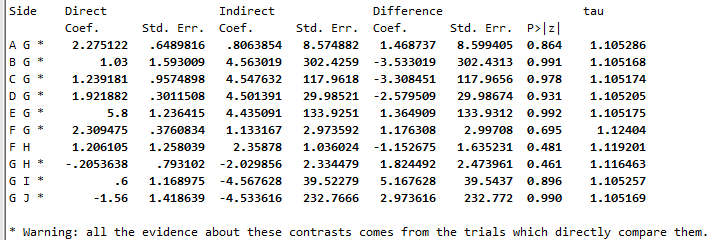


# Supplementary TABLE S7.5 Node-Splitting Results of WHR


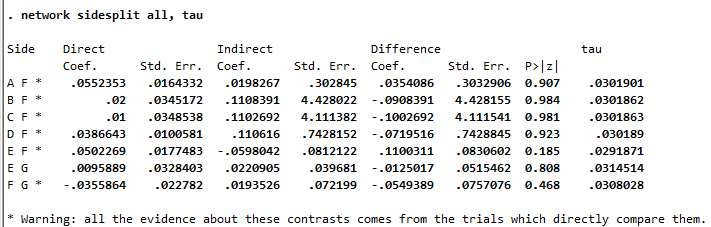


# Supplementary TABLE S7.6 Node-Splitting Results of T


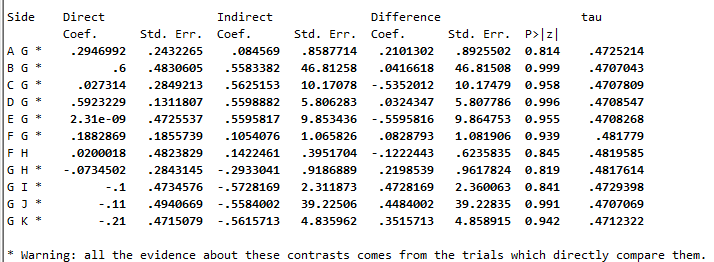


# Supplementary TABLE S7.7 Node-Splitting Results of LH


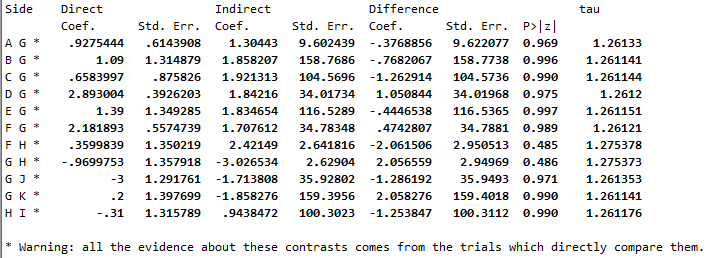


# Supplementary TABLE S7.8 Node-Splitting Results of FSH


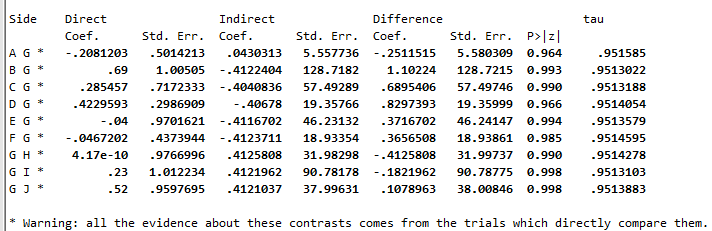


# Supplementary TABLE S7.9 Node-Splitting Results of LH/FSH


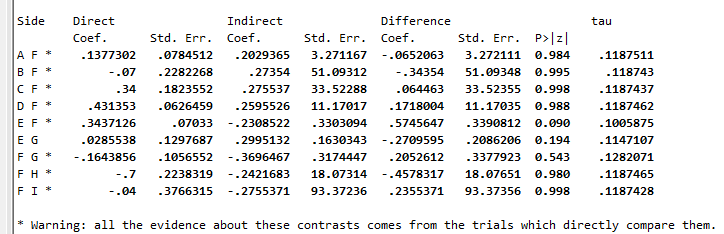


# Supplementary TABLE S7.10 Node-Splitting Results of AFC


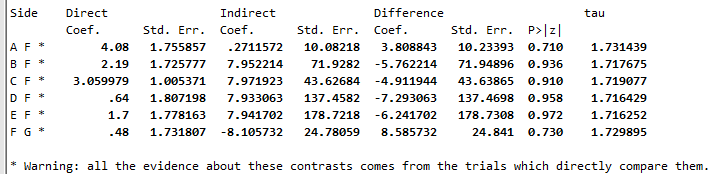


# Supplementary TABLE S7.11 Node-Splitting Results of OV


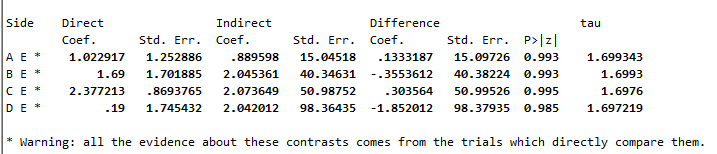


# Supplementary TABLE S8 Loop Inconsistency Factor Test Results in Network Meta-Analysis

| Outcome indicators | Loop | IF | seIF | Z | P | 95%CI | Loop Heterog tau2 |
| --- | --- | --- | --- | --- | --- | --- | --- |
| HOMA-IR | CIAA-CT-EA | 0.427 | 0.266 | 1.609 | 0.108 | (0.00,0.95) | 0.013 |
| FINS | CIAA-CT-EA | 3.238 | 2.257 | 1.435 | 0.151 | (0.00,7.66) | 2.175 |
| FBG | CIAA-CT-EA | 0.569 | 0.409 | 1.389 | 0.165 | (0.00,1.37) | 0.075 |
| BMI | CIAA-CT-EA | 1.044 | 1.863 | 0.560 | 0.575 | (0.00,4.70) | 1.998 |
| WHR | CIAA-CT-EA | 0.014 | 0.013 | 1.105 | 0.269 | (0.00,0.04) | 0.000 |
| T | CIAA-CT-EA | 0.162 | 0.166 | 0.978 | 0 .328 | (0.00,0.49) | 0.016 |
| LH | CIAA-CT-EA | 1.218 | 1.012 | 1.204 | 0.229 | (0.00,3.20) | 0.187 |
| LH/FSH | CIAA-CT-EA | 0.174 | 0.245 | 0.708 | 0.479 | (0.00,0.65) | 0.025 |

# Supplementary FIGURE: Forest Plot of Network Meta-Analysis with Consistency Test


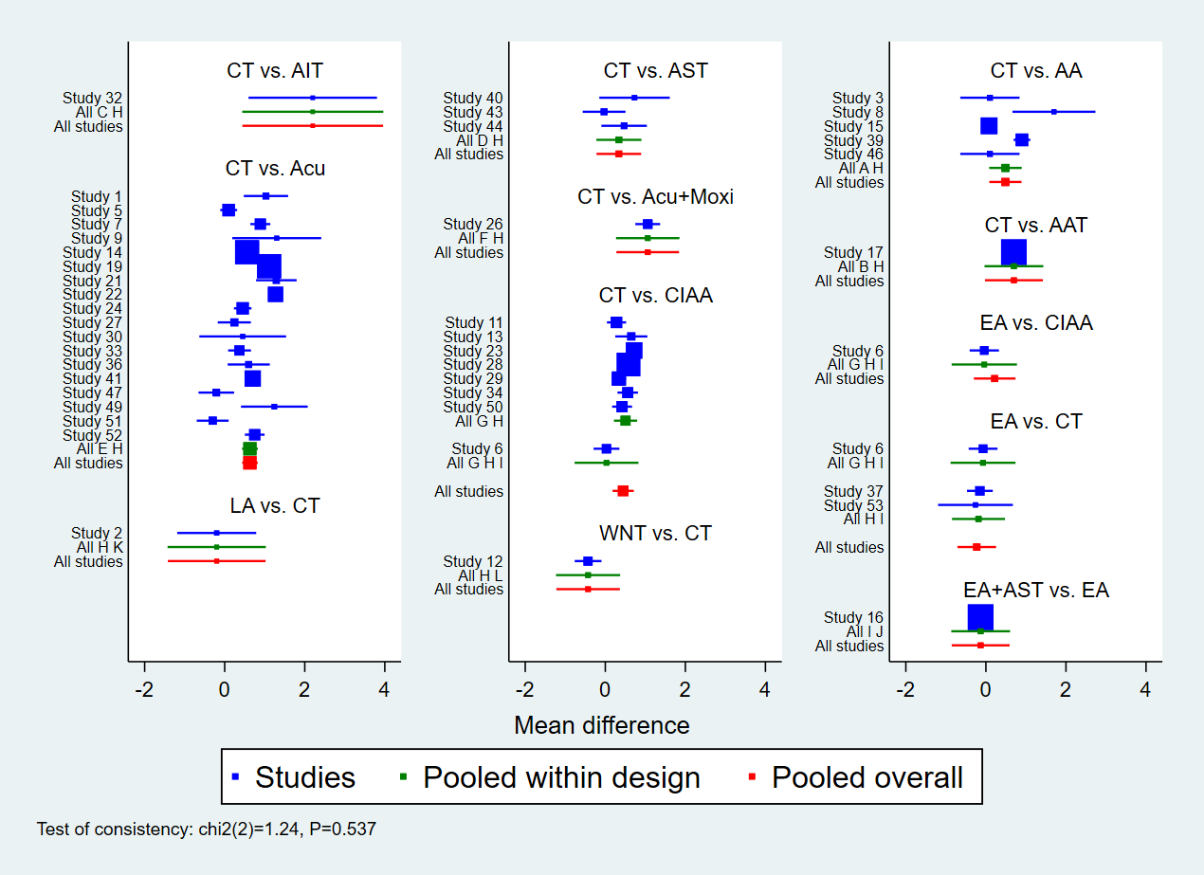


Supplementary FIGURE S1 The forest plot of HOMA-IR.


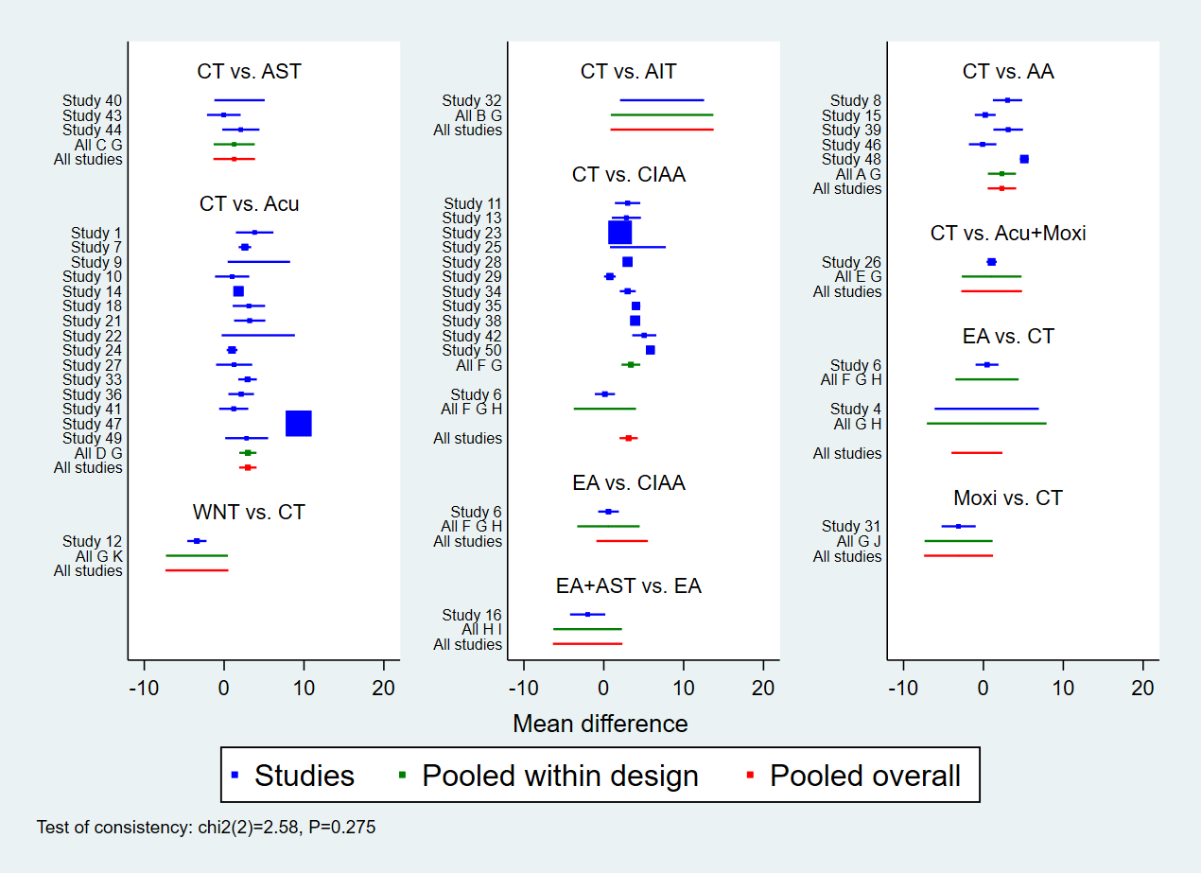


Supplementary FIGURE S2 The forest plot of FINS.


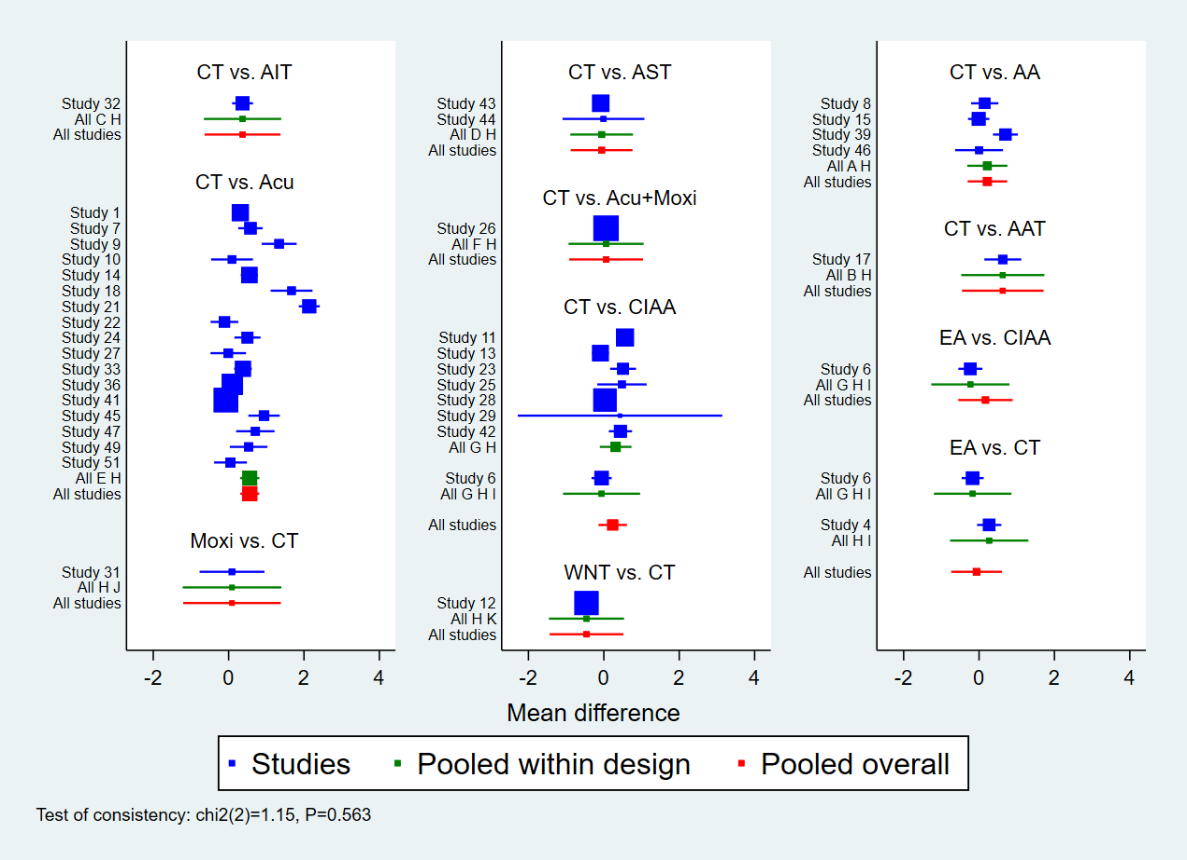


Supplementary FIGURE S3 The forest plot of FBG.


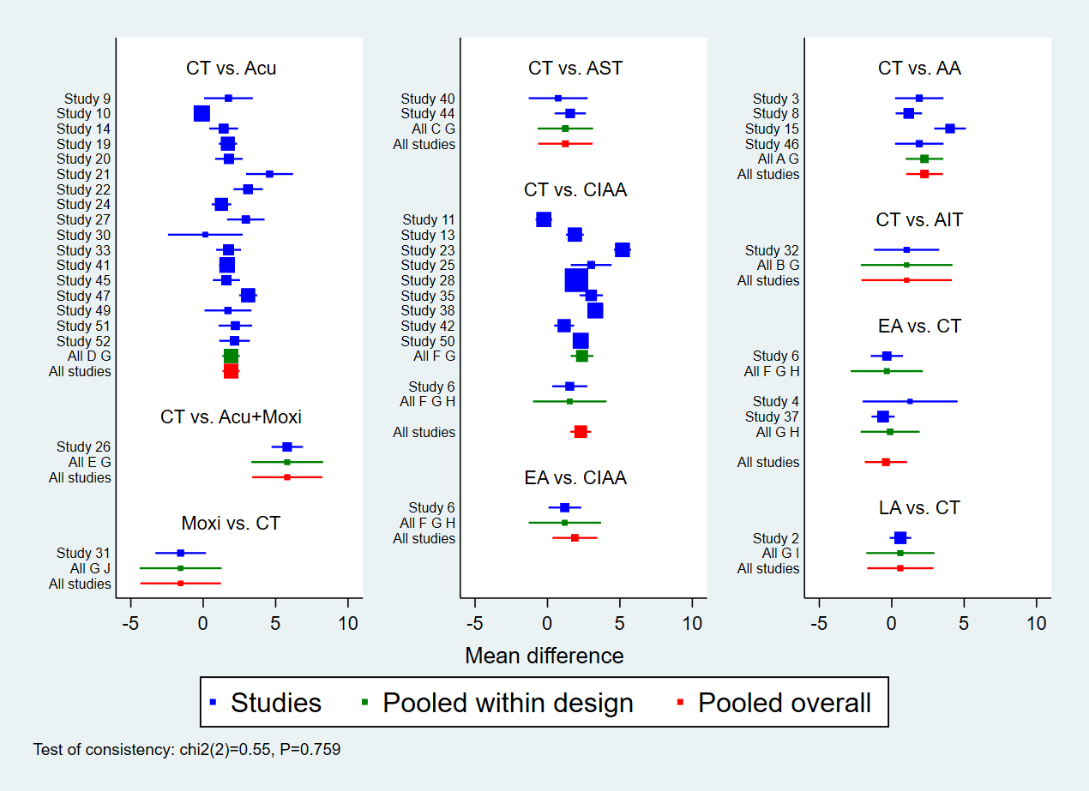


Supplementary FIGURE S4 The forest plot of BMI.


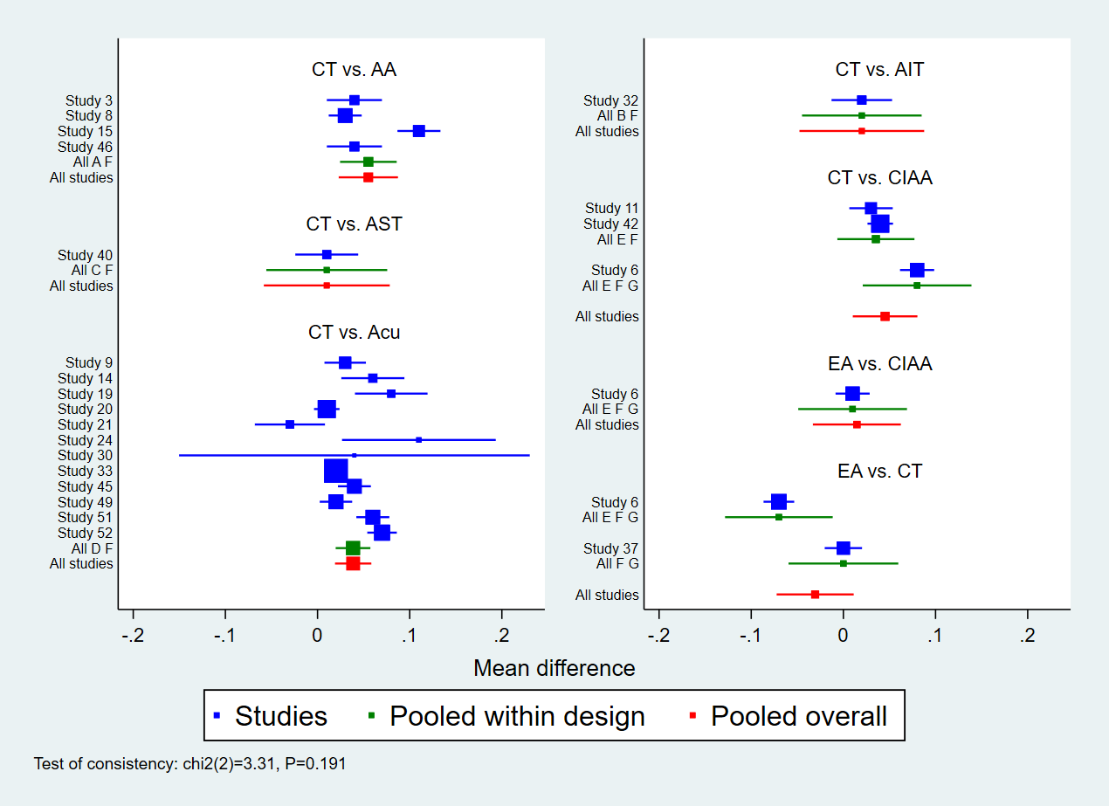


Supplementary FIGURE S5 The forest plot of WHR.


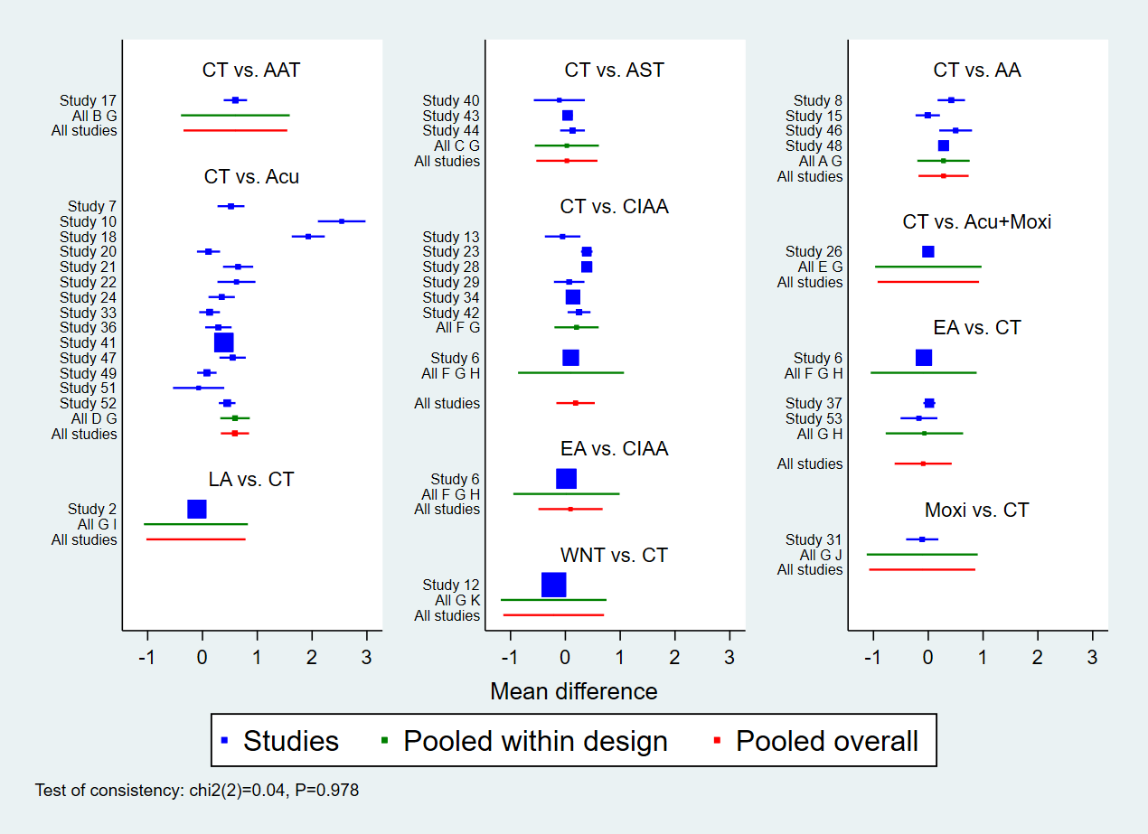


Supplementary FIGURE S6 The forest plot of T.


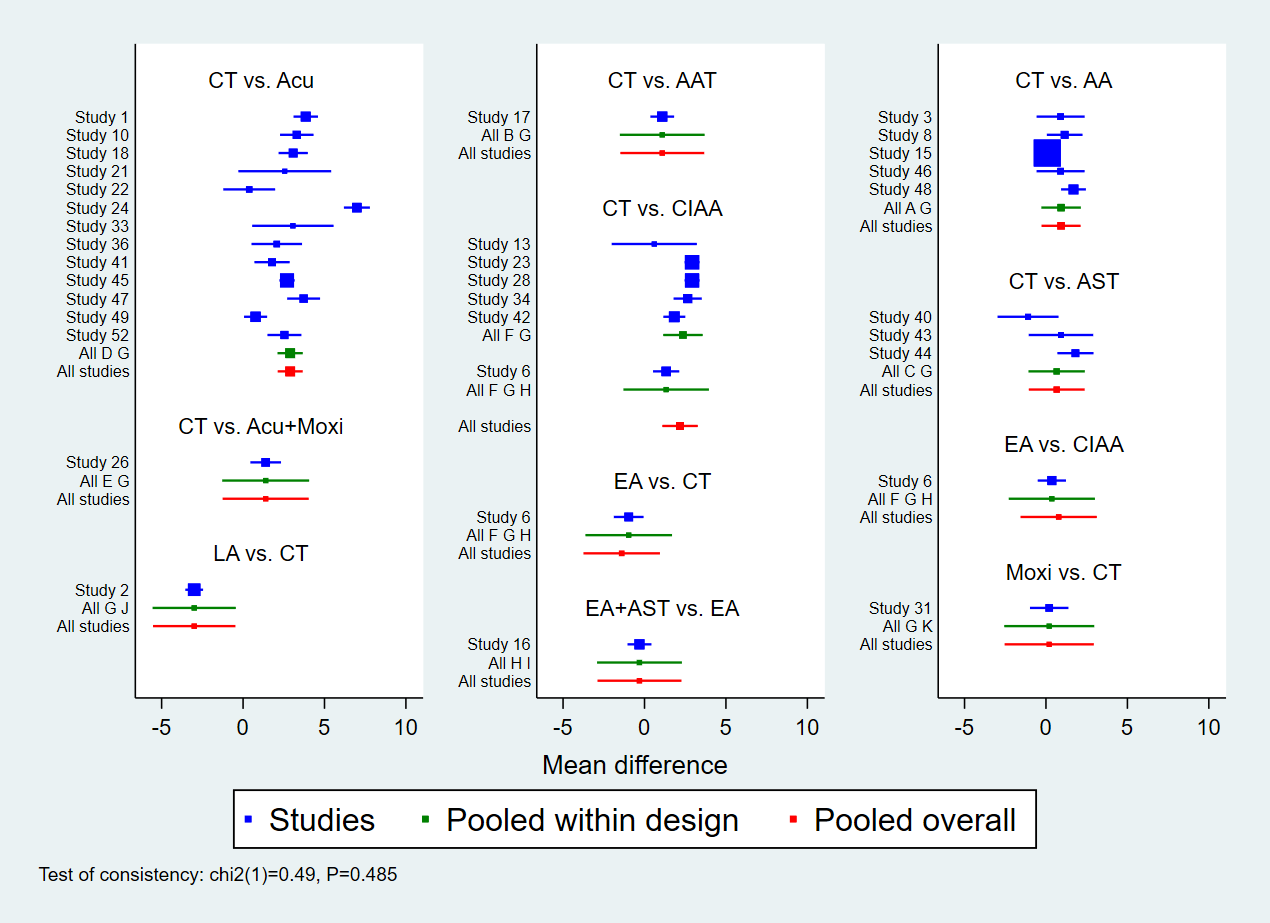


Supplementary FIGURE S7 The forest plot of LH.


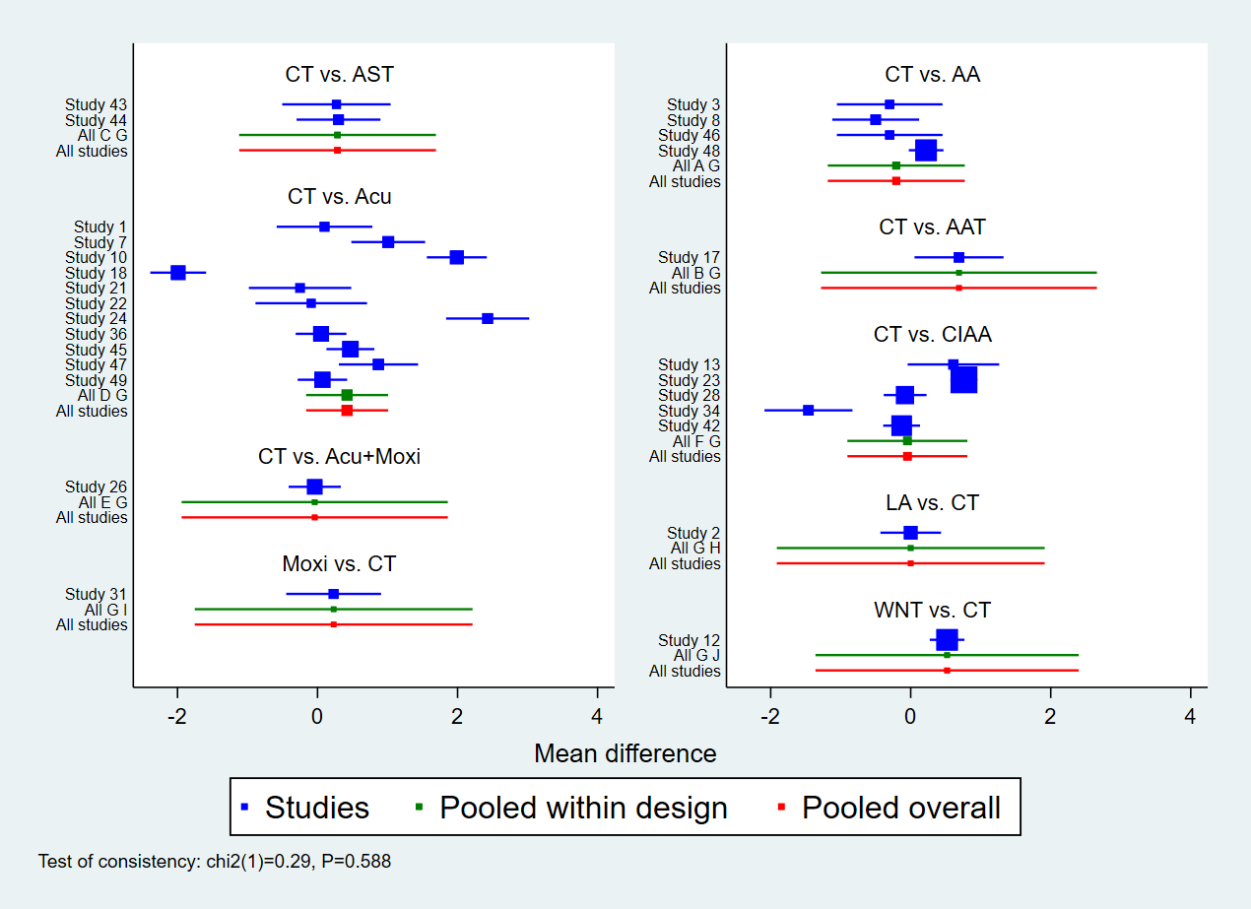


Supplementary FIGURE S8 The forest plot of FSH.


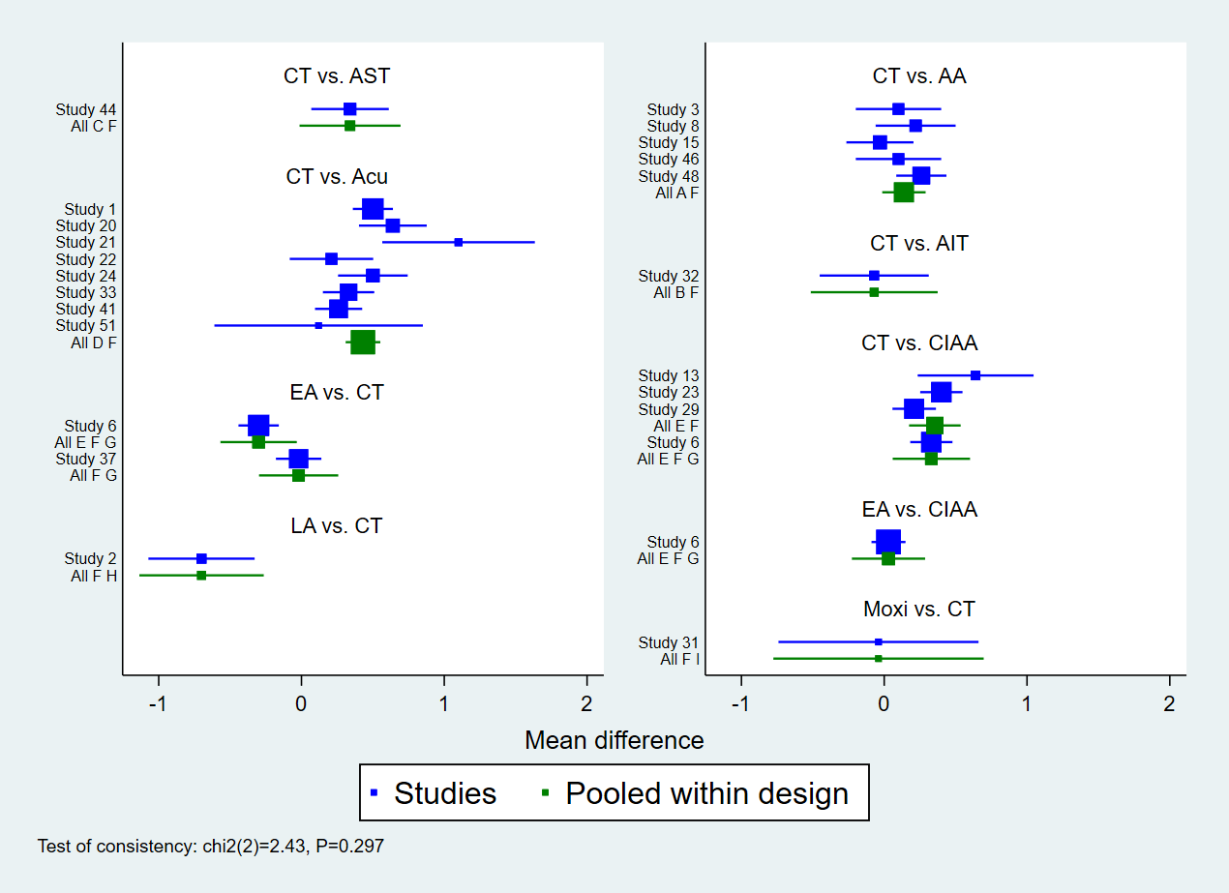


Supplementary FIGURE S9 The forest plot of LH/FSH.


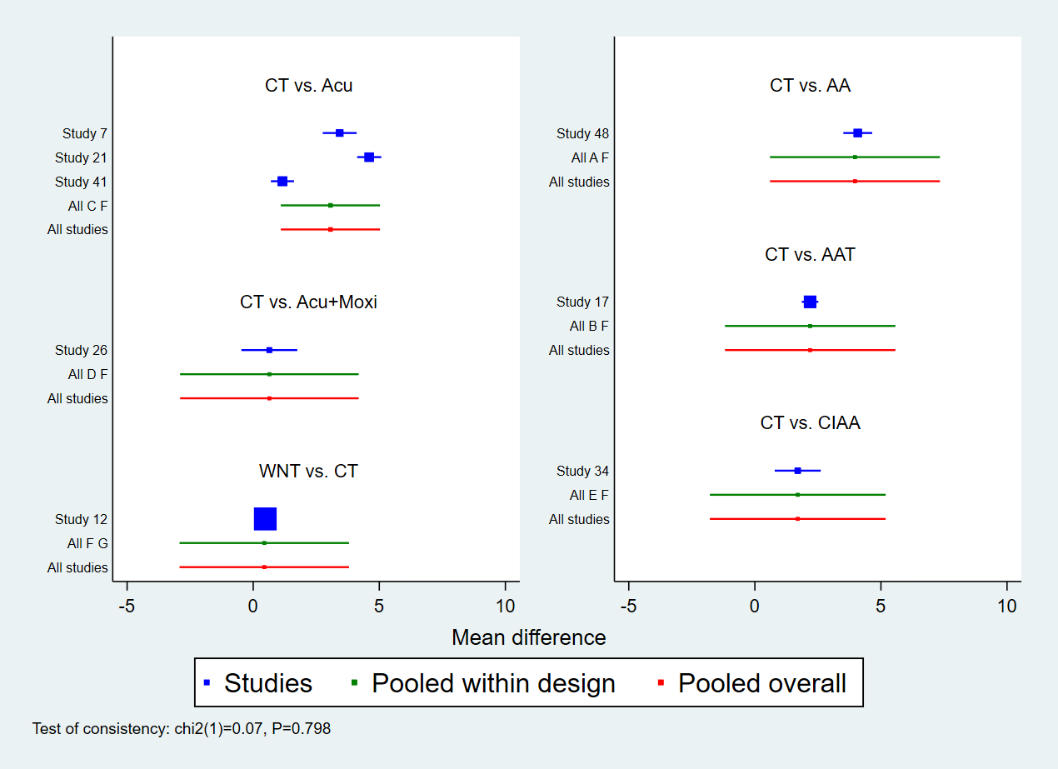


Supplementary FIGURE S10 The forest plot of AFC.


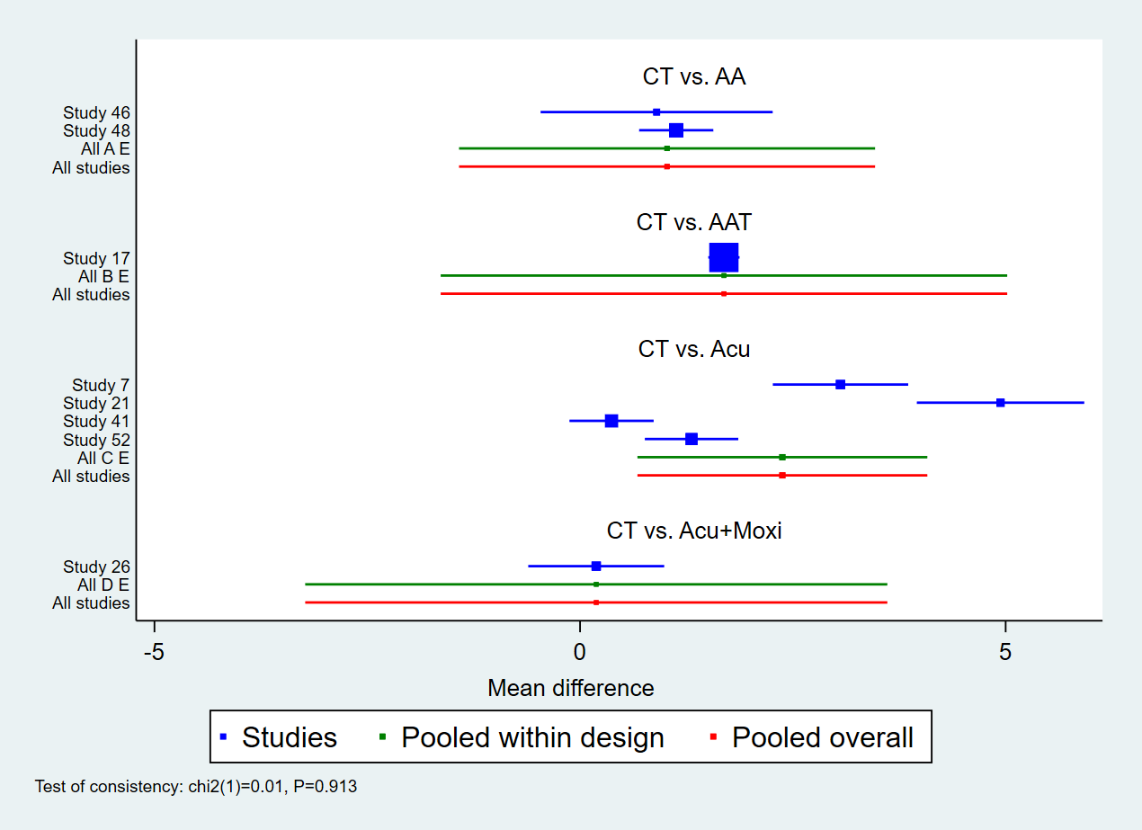


Supplementary FIGURE S11 The forest plot of OV.

# **Reference**

1. WU T, XU G, HONG X, FAN H, ZENG J, LIU Y, HU J, LIANG F, YANG J, CHEN J. Acupuncture for hormonal readiness and gut microbiota in obese polycystic ovary syndrome: an open-label, randomized controlled trial. *Front Endocrinol* (2024) 15: doi: 10.3389/fendo.2024.1509152

2. El-Shamy FF, El-kholy SS, El-Rahman MMA. Effectiveness of laser acupoints on women with polycystic ovarian syndrome: a randomized controlled trial. *J Lasers Med Sci* (2018) 9:113–120. doi: 10.15171/jlms.2018.22

3. ZHENG Y-H, WANG X-H, LAI M-H, YAO H, LIU H, MA H-X. Effectiveness of abdominal acupuncture for patients with obesity-type polycystic ovary syndrome: a randomized controlled trial. *J Altern Complement Med* (2013) 19:740–745. doi: 10.1089/acm.2012.0429

4. Muharam R, Ph.D., Srilestari A, Mihardja H, Juvanni Callestya L, Kemal Harzif A. Combination of electroacupuncture and pharmacological treatment improves insulin resistance in women with polycystic ovary syndrome: Double-blind randomized clinical trial. *Int J Reprod Biomed* (2022) 20:289–298. doi: 10.18502/ijrm.v20i4.10900

5. WEN Q, HU M, LAI M, LI J, HU Z, Quan K, Liu J, Liu H, Meng Y, Wang S, et al. Effect of acupuncture and metformin on insulin sensitivity in women with polycystic ovary syndrome and insulin resistance: a three-armed randomized controlled trial. *Hum Reprod (oxf Engl)* (2021) 37:542–552. doi: 10.1093/humrep/deab272

6. CAI X, LI Y, WANG J, CAO J. Clinical Observation of Electroacupuncture and Acupoint Catgut-Embedding Therapy in the Treatment of Obese Polycystic Ovary Syndrome. *Guangming Journal of Chinese Medicine* (2016) 31:538–541.

7. CHAI H, LU F, YU J, ZHENG Y, DU C. Effect of acupuncture and Jianpi Bushen Decoction on ovarian function and metabolic disorders in patients with PCOS. *Journal of Hainan Medical University* (2021) 27:111–115, 120. doi: 10.13210/j.cnki.jhmu.20200715.002

8. DUN J, TIAN L, WANG F. Effect of Abdominal Acupuncture Combined with Metformin on Insulin Resistance of Puberty Phlegm Dampness Polycystic Ovary Syndrome. *Clinical Journal of Traditional Chinese Medicine* (2018) 30:1236–1239. doi: 10.16448/j.cjtcm.2018.0378

9. GU Y, XU F, HE X. Evaluation the effect of acupuncture improves glycolipid metabolism disorder in patients with polycystic ovary syndrome. *Journal of Li-shizhen Traditional Chinese Medicine* (2019) 30:2431–2433.

10. HA H. Analysis of Endocrine Status in Patients with Polycystic Ovary Syndrome Treated with Pinellia and Coptis Decoction Combined with Syndrome Differentiation-Based Acupuncture Point Selection. *Maternal and Child Health Care of China* (2019) 34:692–695.

11. JIANG M, ZHU J, CHEN P. Combination of catgut embedment in acupoints and Chinese medicine on insulin resistance and serum leptin levels of patients with polycystic ovarian syndrome. *Shanghai Journal of Traditional Chinese Medicine* (2015) 49:52–54. doi: 10.16305/j.1007-1334.2015.02.013

12. JIANG X, CHU C, YAN D, XUE X, YANG Y, ZHANG L. Effect of Warm Acupuncture Treatment Combined with Strengthening Spleen and Expelling Phlegm Method in Treatment of Normal Body Weight Patients with Polycystic Ovary Syndrome and Tts Effects on Sexual Hormones and Insulin Resistance. *Medical & Pharmaceutical Journal of Chinese People’s Liberation Army* (2017) 29:105–109.

13. JIANG X, MENG J. Observation of acupoint catgut implantation combined with metformin hydrochloride tablets on renal deficiency and phlegm- damp type polycystic ovarian syndrome with insulin resistance. *Hebei Journal of Traditional Chinese Medicine* (2014) 36:171–174.

14. JIANG Y, YAO H, PAN J, WANG X. Clinical study of Tongbu Qijing Acupuncture combined with metformin hydrochloride tablet in the treatment of polycystic ovary syndrome combined with insulin resistance of kidney-deficiency phlegm dampness type. *International Journal of Traditional Chinese Medicine* (2023) 45:813–817. doi: 10.3760/cma.j.cn115398-20220628-00363

15. LAI M, MA H, LIU H, SONG X, DING T, ZHENG Y. Clinical Observation of Abdominal Acupuncture in the Treatment of 60 Cases of Polycystic Ovary Syndrome with Spleen-Kidney Yang Deficiency Pattern. *Jiangsu Journal of Traditional Chinese Medicine* (2012) 44:53–54.

16. LI L, ZHANG Y, WANG J. Effect of Electroacupuncture Combined with Auricular Pressure on Serum Hormones and Insulin Levels in Patients with Polycystic Ovary Syndrome. *Journal of Hunan University of Chinese Medicine* (2015) 35:52–55.

17. LIU B, ZHANG C, DONG Q, WEI X, WANG J. Efficacy of Acupoint Application Combined with Spleen-regulating and Kidney-tonifying Therapy on Treatment of Polycystic Ovary Syndrome and Its Impact on Pregnancy Rate. *World Journal of Integrated Traditional and Western Medicine* (2024) 19:2458–2462. doi: 10.13935/j.cnki.sjzx.241221

18. LIU X. The Effect of Traditional Chinese Medicine Pattern Differentiation and Acupoint Selection Combined with Pinellia Decoction for Clearing the Heart on Insulin Sensitivity and Endocrine Function in Patients with Polycystic Ovary Syndrome. *Practical Clinical Journal of Integrated Traditional Chinese and Western Medicine* (2017) 17:36–37. doi: 10.13638/j.issn.1671-4040.2017.03.021

19. MAO M, LIN L. Clinical Observation of Tongyuan Acupuncture for the Treatment of Polycystic Ovary Syndrome with Phlegm-dampness Due to Spleen Deficiency Syndrome. *Journal of Guangzhou University of Traditional Chinese Medicine* (2021) 38:2138–2145. doi: 10.13359/j.cnki.gzxbtcm.2021.10.016

20. PENG Y, CONG J, HU N, CHANG H, LV N, YANG X. Acupuncture with Resolving Phlegm Therapy in the Treatment of Polycystic Ovary Syndrome: Evaluation of Clinical Efficacy. *Journal of Clinical Acupuncture and Moxibustion* (2017) 33:5–8.

21. QUAN C, ZHANG Z, HE X, CAO H, LIU W, LEI M. Acupuncture Combined with Metformin in the Treatment of 60 Cases of Polycystic Ovary Syndrome with Spleen Deficiency and Phlegm-Dampness Pattern. *Hunan Journal of Traditional Chinese Medicine* (2021) 37:73–75, 118. doi: 10.16808/j.cnki.issn1003-7705.2021.11.023

22. SU J, LI Y, TIAN L, SHAO J. Treating polycystic ovary syndrome with obesity by acupuncture combined with tonifying kidney,reducing phlegm and dredging collateral. *Tianjin Journal of Traditional Chinese Medicine* (2013) 30:274–276.

23. SONG N, MA Z, CHENG C. Clinical Efficacy of Acupoint Embedding Combined with Ethinylestradiol and Cyproterone Acetate Tablets in the Treatment of Polycystic Ovary Syndrome. *Shenzhen Journal of Integrated Traditional Chinese and Western Medicine* (2024) 34:18–21. doi: 10.16458/j.cnki.1007-0893.2024.17.005

24. SU Y, DONG W, GUO W, ZHU J, JIANG M. Efficacy observation of abdominal circling needling method on polycystic ovary syndrome with syndrome of phlegm-dampness. *Shanxi Journal of Traditional Chinese Medicine* (2025) 41:29–31. doi: 10.20002/j.issn.1000-7156.2025.06.011

25. TAO L, LONG Y, SANG X, ZHANG Y, CHEN X, NIU X, LIN X. Effects of point catgut-embedding therapy combined Chinese medicinal herbs of invigorating spleen to remove phlegm on insulin resistance and serum adiponectin level in obese patients with polycystic ovary syndrome. https://qikan.cqvip.com/Qikan/Article/ReadIndex?id=27126756&info=%2bRyv0bHc3H0UR8ZX6vWDBj3qkUHwHyaO7Q4yjshxFtM%3d [Accessed October 7, 2025]

26. WANG W, LIU P, SHOU Y, ZHANG K, YUAN L, LI B, GENG Z, ZHANG B. Clinical study on acupuncture and thunder-fire Moxibustion therapy for spleen and kidney deficiency-type polycystic ovary syndrome. *Journal of China Prescription Drug* (2021) 19:158–161.

27. WANG C, SUN X, DING C, SHEN Y. Effects of Modified Cangfu Daotan Decoction Combined with Acupuncture on Glucose and Lipid Metabolism and Ovulation Rates in Obese Patients with Polycystic Ovary Syndrome. *Modern Journal of Integrated Traditional Chinese and Western Medicine* (2016) 25:4056–4058.

28. WANG L, CHEN J, YU J, SUN Y. Clinical Study of Acupuncture Point Implantation Combined with Metformin in the Treatment of Obesity-Associated Polycystic Ovary Syndrome with Insulin Resistance: A 40-Case Cohort. *Jiangsu Journal of Traditional Chinese Medicine* (2025) 57:40–44. doi: 10.19844/j.cnki.1672-397X.2025.03.011

29. WANG Q, WANG R. Clinical Observation of Acupoint Catgut Embedding Combined with Shoushen Tiaojing Decoction and Metformin in Treating Obese PCOS. *Clinical Journal of Traditional Chinese Medicine* (2023) 35:1161–1165. doi: 10.16448/j.cjtcm.2023.0625

30. WANG Y, XING Y, XING Y, YANG Y, LIU Y. Clinical efficacy observation of Bushen Huatan Decoction combined with acupuncture in the treatment of obese polycystic ovary syndrome. *Chinese Journal of Family Planning & Gynecotokology* (2022) 14:75–80.

31. WANG Y, XU J, HU Z, WANG S, XIONG Z, BAI Z, GU L. Observations on the Therapeutic Effect of Du meridian Moxibustion on Polycystic Ovarian Syndrome of Spleen-kidney Yang Deficiency Type. *Shanghai Journal of Acupuncture and Moxibustion* (2015) 34:35–37. doi: 10.13460/j.issn.1005-0957.2015.01.0035

32. WU J, ZHANG S, TANG L, ZU Y, ZHANG X, ZHU D. Clinical Observation of Acupoint Injection Improving Glycometabolism in Patients with Polycystic Ovary Syndrome of Kidney Deficiency and Blood Stasis. *Modernization of Traditional Chinese Medicine and Materia Medica-World Science and Technology* (2025) 27:1982–1989.

33. XU R, JI L. Observational Study on the Efficacy of Acupuncture Therapy for Obese Patients with Polycystic Ovary Syndrome. *Modern Medicine and Health Research Electronic Journal* (2024) 8:92–94.

34. XU Y, MI H, YANG Y. Effect of acupoint catgut embedding therapy on metabolism and hormone levels in patients with polycystic ovary syndrome. *Chinese Journal of General Practice* (2024) 22:665–668. doi: 10.16766/j.cnki.issn.1674-4152.003475

35. YANG W. Clinical Observation on the Combined Use of Cangfu Daotan Decoction and Acupoint Implantation Therapy for Obesity-Type Polycystic Ovary Syndrome. *Journal of Practical Traditional Chinese Medicine* (2021) 37:367–368.

36. YANG X. Clinical Study on Shouwu Jiangqi Decoction Combined with Acupuncture and Moxibustion in the Treatment of Polycystic Ovary Syndrome of Kidney Deficiency and Phlegm Stasis. *Guangming Journal of Chinese Medicine* (2022) 37:2369–2373.

37. YAO M, DING D, ZHOU W, HUANG W, XU X. Effect of Acupuncture and Moxibustion on Patients’ Anxiety in Treatment of Obesity Type Polycystic Ovary Syndrome. *Acta Chinese Medicine* (2018) 33:2043–2048. doi: 10.16368/j.issn.1674-8999.2018.10.484

38. YE L, YANG Z, JIANG Y, CHEN Y, SHAN L, QIAN C. Traditional Chinese Drugs for Strengthening Spleen and Resolving Phlegm and Points Embedding Therapy for Glucolipid Metabolism of Patients with Polycystic Ovary Syndrome. *Chinese Archives of Traditional Chinese Medicine* (2018) 36:1634–1636. doi: 10.13193/j.issn.1673-7717.2018.07.025

39. YIN T, LI M, CHEN D, LI X, QIAN H. The Effect of Abdominal Fat on Insulin Resistance in Patients with Polycystic Ovary Syndrome. *Shanxi Journal of Traditional Chinese Medicine* (2016) 32:36, 38.

40. ZHANG W, LIU X, WANG D, LIAO Y, CHEN T, LIU Y. Clinical observation of auricular point sticking and Banxia Xiexin Decoction on polycystic ovary syndrome combined with insulin resistance of stomach heat and spleen deficiency. *Hebei Journal of Traditional Chinese Medicine* (2025) 47:996–1001.

41. ZHANG Y. Effects of Acupuncture and Moxibustion Therapy on Adiponectin, Leptin and Insulin Resistance in Obese Patients with Polycystic Ovary Syndrome. *Acta Chinese Medicine* (2017) 32:2259–2262. doi: 10.16368/j.issn.1674-8999.2017.11.588

42. ZHAO Y. Clinical Observation of Combined Traditional Chinese Medicine and Acupoint Implantation Therapy for Polycystic Ovary Syndrome. *Chinese Science and Technology Journal Database (Abstract Edition) - Medicine and Health* https://qikan.cqvip.com/Qikan/Article/ReadIndex?id=1000003339610&info=c4socuTxv6e%2ftbPqnbQYKcqp%2bUl4widIgv1BauJQB4SADUAprKZ2UA%3d%3d [Accessed October 7, 2025]

43. ZHU S, LIU X, LIAO Y, REN Y, LIU Y. Clinical Efficacy of Chaishao Duonang Decoction Combined with Auricular Acupressure in the Treatment of Polycystic Ovary Syndrome with Kidney Deficiency and Liver Stagnation Syndrome and Effect on Safety. *World Journal of Integrated Traditional and Western Medicine* (2025) 20:1354–1360. doi: 10.13935/j.cnki.sjzx.250713

44. LI Y, HAO S, ZHANG C, SUN M, ZHANG M, HOU L. Clinical observation of auricular acupoint pressing for treatment of puberty polycystic ovary syndrome. *Modern Journal of Integrated Traditional Chinese and Western Medicine* (2018) 27:3877–3879, 3906.

45. WANG J, MA Y, DU W, WANG B, WU L. Clinical study of influence of Metformin combined with acupuncture on glucolipid metabolism and adipokines in obese polycystic ovarian syndrome. *China Modern Medicine* (2020) 27:69–72.

46. LAI M, MA H, YAO H, LIU H, SONG X, HUANG W, WU X. Effect of Abdominal Acupuncture Therapy on the Endocrine and Metabolism in Obesity-type Polycystic Ovarian Syndrome Patients. *Acupuncture Research* (2010) 35:298–302. doi: 10.13702/j.1000-0607.2010.04.013

47. HUANG C, WEI L. Observation on the therapeutic effect of acupuncture at Zhuang medicine’s circular points based on Zhuang medicine’s food track theory in the treatment of PCOS with insulin resistance. *Guangxi Journal of Traditional Chinese Medicine* (2025) 48:57–61.

48. CHEN W, CHEN H, QIN M, CAI W. Clinical Observation of Abdomen Acupuncture of Lingnan Flying Needle Therapy in Treating Polycystic Ovary Syndrome of Phlegm-dampness Due to Spleen Deficiency Syndrome. *Journal of Guangzhou University of Traditional Chinese Medicine* (2021) 38:2151–2157. doi: 10.13359/j.cnki.gzxbtcm.2021.10.018

49. LI Y, PENG J, QU Y, TIAN Y, XIANG J, ZHANG J, DENG X. Clinical Effect Observation of “Shuanggu Yitong″ Acupuncture Treating Obese Polycystic Ovary Syndrome. *Journal of Jianghan University (Natural Science Edition)* (2024) 52:20–27. doi: 10.16389/j.cnki.cn42-1737/n.2024.02.003

50. MA G, HU Z, SHI Y, WANG Y, HE J, ZHANG A, DONG H. Effect of Acupoint Thread-embedding Combined with Metformin on Glucose and Lipid Metabolism in Obese Polycystic Ovary Syndrome Patients. *Shanghai Journal of Acupuncture and Moxibustion* (2020) 39:1123–1127. doi: 10.13460/j.issn.1005-0957.2020.09.1123

51. ZHAO Y, HU L, WANG C, WANG X, DU X, SHAO H, SUN J, YU R. The study of treatment with compound acupuncture-metformin on the outcome of ovulation induction with Clomiphene in the patients with polycystic ovarian syndrome. *Jiangxi Medical Journal* (2007)1089–1092.

52. ZHANG X, WANG H, XIE C. Clinical observation on therapeutic effect of acupuncture combined with Western medicine on obese patients with polycystic ovary syndrome. *Journal of Hubei University of Chinese Medicine* (2022) 24:86–88.

53. YU L, CAO L, XIE J, SHI Y, ZHOU L, HE T, CHU X. Efficacy and mechanism of electroacupuncture on insulin resistant polycystic ovary syndrome. *Chinese Acupuncture & Moxibustion* (2020) 40:379–383. doi: 10.13703/j.0255-2930.20190903-k0003
